# Phytochemical Analysis of Plant *Nanophyton iliense* U.P. Pratov from Kazakhstan Using LC-MS

**DOI:** 10.3390/molecules31060918

**Published:** 2026-03-10

**Authors:** Kudaibergenova Moldir K., Datkhayev Ubaidilla M., Bharathi Avula, Kumar Katragunta, Kiran Kumar Tatapudi, Jennyfer A. Aldana-Mejía, Ikhlas A. Khan, Akhtayeva Nursulu Z., Mukhametzhan Ayala S., Kiyekbayeva Lashyn N., Samir A. Ross

**Affiliations:** 1 School of Pharmacy, Kazakh National Medical University named after S.D. Asfendiyarov, st. Tole Bi 94, Almaty 050000, Kazakhstan; manali89@mail.ru (K.M.K.); u.datxaev@mail.ru (D.U.M.); 2 National Centre for Natural Products Research, School of Pharmacy, University of Mississippi, Oxford, MS 38677, USA; bavula@olemiss.edu (B.A.); kkatragu@olemiss.edu (K.K.); kktatapu@olemiss.edu (K.K.T.); jaaldana@olemiss.edu (J.A.A.-M.); ikhan@olemiss.edu (I.A.K.); 3 Division of Pharmacognosy, Department of BioMolecular Sciences, School of Pharmacy, University of Mississippi, Oxford, MS 38677, USA; 4 Department of Biodiversity and Bioresources, Faculty of Biology and Biotechnology, Al-Farabi Kazakh National University, Almaty 050000, Kazakhstan; akhtaeva@mail.ru; 5 International School of Medicine, Kazakh National Medical University named after S.D. Asfendiyarov, st. Tole Bi 94, Almaty 050000, Kazakhstan; amukhametzhan2007@gmail.com

**Keywords:** *Nanophyton iliense*, phytochemical profiling, LC-DAD-QToF-MS, hydroxycinnamic acid amides, flavonoids, Kazakhstan flora

## Abstract

To date, the phytochemical composition of the aerial parts of *Nanophyton iliense* U.P. Pratov has not been comprehensively investigated. In the present study, qualitative metabolite profiling of the methanolic extract of the aerial parts was performed using liquid chromatography coupled with diode-array detection and quadrupole time-of-flight mass spectrometry (LC-DAD-QToF-MS) operating in both positive and negative electrospray ionization modes. A total of 81 metabolites were tentatively identified based on accurate mass measurements, MS/MS fragmentation patterns obtained in all-ion MS/MS mode, and comparison with previously reported literature data. The detected compounds included hydroxycinnamic acid amides, phenolic acids, flavonoids (including glycosides), amino acids, organic acids, sulfated derivatives, and nucleosides. Among them, the flavonoid narcissin (isorhamnetin-3-O-rutinoside) was isolated from the extract, and its structure was confirmed by ^1^H and ^13^C NMR spectroscopy supported by COSY, HSQC, and HMBC experiments. Additionally, a compound with the molecular formula C_17_H_14_O_5_ was detected; however, its structure could not be conclusively established based on the available spectroscopic data and is therefore reported as an unidentified metabolite. The present study provides the first systematic qualitative characterization of the metabolite profile of *N. iliense* and establishes a foundation for future quantitative and bioactivity-oriented investigations of this species.

## 1. Introduction

Plants inhabiting arid and saline environments are recognized as important sources of structurally diverse secondary metabolites that contribute to stress tolerance, ecological adaptation, and potential biological activity [1,2,3,4,5,6,7]. Halophytic and desert-adapted taxa are particularly known for the accumulation of phenolic compounds, flavonoids, hydroxycinnamic acid derivatives, and related phenylpropanoid metabolites involved in antioxidative defense and environmental stress responses [2,3,4,5]. These metabolites are closely associated with phenylpropanoid metabolism and play key roles in plant resilience under extreme climatic conditions [4,5,6].

The genus *Nanophyton iliense* (Amaranthaceae, Caryophyllales) comprises slow-growing desert shrubs distributed predominantly in arid and temperate regions of Southern European Russia and Central Asia. Species of this genus are adapted to saline and drought-prone habitats, suggesting the presence of specialized metabolic pathways supporting stress tolerance. Despite their ecological significance, phytochemical studies of *N. iliense* species remain limited. Phenylpropanoid amides and related phenolic constituents have previously been reported in *Nanophyton erinaceum* [8], and phenolic metabolites are widely distributed within Amaranthaceae species [1,2,9]. However, comprehensive metabolite profiling of *N. iliense* using high-resolution analytical platforms has not yet been systematically performed.

Liquid chromatography coupled with high-resolution mass spectrometry (LC–HRMS) has become one of the most powerful analytical approaches for untargeted phytochemical profiling of complex plant matrices [10,11,12,13,14,15,16]. The combination of chromatographic separation with accurate mass detection enables sensitive characterization of both primary and secondary metabolites across a broad polarity range [13,14,15]. In particular, quadrupole time-of-flight mass spectrometry (LC-DAD-QToF-MS) provides exact mass measurements, isotopic pattern analysis, and high-resolution MS/MS fragmentation, facilitating confident tentative annotation of metabolites, even in the absence of authentic standards [12,13,17,18].

The integration of LC-DAD-QToF-MS with diode array detection (DAD) further enhances metabolite annotation by providing complementary UV–VIS spectral information, which is especially valuable for phenolic acids, flavonoids, and coumarin derivatives [11,12,13]. Such hyphenated analytical platforms are widely applied in phytochemical investigations of medicinal and stress-adapted plant species [13,14,15,17,18].

Secondary metabolites such as hydroxycinnamic acid amides (HCAAs), flavonoids, and glycosylated phenolics play essential roles in plant defense and adaptation to abiotic stress [2,3,4,5]. HCAAs, formed through conjugation of hydroxycinnamic acids with biogenic amines via the phenylpropanoid pathway, are increasingly recognized as stress-associated metabolites [2,5] and exhibit characteristic MS/MS fragmentation patterns [19,20,21]. Similarly, flavonoids and phenolic acids, including caffeoylquinic and coumaric acid derivatives, are widely distributed in desert plants and represent important chemotaxonomic constituents [19,20,22,23].

In this context, the present study aims to perform a comprehensive phytochemical profiling of the aerial parts of *N. iliense* using LC-DAD-QToF-MS operating in both positive and negative electrospray ionization modes. In addition to HRMS-based metabolite annotation, selected compounds were isolated and structurally characterized using NMR spectroscopy to confirm their identities. The study focuses on the identification of primary and secondary metabolites based on accurate mass measurements, retention behavior, MS/MS fragmentation patterns, and spectroscopic data, following established annotation strategies [17,18].

## 2. Results

### 2.1. LC-QToF-MS Analysis of the Aerial Parts of Nanophyton iliense

Qualitative metabolite profiling of the methanolic extract of the aerial parts of *N. iliense* was performed using LC-QToF-MS operating in both positive and negative electrospray ionization modes. A total of eighty-one metabolites were tentatively annotated based on accurate mass measurements, isotopic pattern consistency, MS/MS fragmentation data, retention behavior on a reversed-phase column, and comparison with previously reported literature and spectral databases. Compound annotation followed established high-resolution MS-based identification principles [15,16,24]. The identified metabolites were classified into hydroxycinnamic acid amides (HCAAs), phenolic acids, flavonoids (including glycosides), amino acids and derivatives, organic acids, sulfated compounds, and additional constituents (Table 1).

#### 2.1.1. Hydroxycinnamic Acid Amides (**1**–**10**)

Ten hydroxycinnamic acid amides (HCAAs) were tentatively identified, predominantly in positive ionization mode. HCAAs are phenylpropanoid-derived conjugates of hydroxycinnamic acids and biogenic amines and are commonly associated with stress adaptation in higher plants [2,3,4,25]. Phenylpropanoid amides have previously been reported in *Nanophyton* species [8], supporting their occurrence in *N. iliense*.

Feruloyl derivatives exhibited a diagnostic fragment ion at *m*/*z* 177.0547–177.0548 corresponding to a feruloyl-derived fragment ion (*m*/*z* 177.0547; [C10H9O3]^+^), whereas protonated ferulic acid is observed at *m*/*z* 195.0652 (Table 1). Additional fragments at *m*/*z* 145.028–145.029 and 117.033–117.034 were consistent with sequential neutral losses, in agreement with reported LC–MS/MS behavior of hydroxycinnamic acid–polyamine conjugates [19,20,21].

For example, a chromatographic peak at 26.04 min showed a precursor ion at *m*/*z* 314.1388 [M + H]^+^ and fragment ions at *m*/*z* 177.0548, 145.0286, and 117.0335, supporting its tentative annotation as feruloyltyramine. Additional feruloyl-containing conjugates were assigned based on similar fragmentation patterns (Table 1).

#### 2.1.2. Phenolic Compounds (**11**–**44**)

Phenolic acids, coumarin derivatives, and flavonoid glycosides were detected in both ionization modes. Phenolic metabolites derived from the phenylpropanoid pathway are characteristic of stress-adapted plant species [2,3,4].

For several low-molecular-weight phenolic acids that did not produce informative MS/MS fragments under the applied all-ion conditions (e.g., cinnamic acid), tentative annotation was based on accurate mass measurement, retention behavior on reversed-phase chromatography, and comparison with previously reported HRMS data [22,23].

Chlorogenic acid isomers were tentatively identified in negative ion mode based on diagnostic fragment ions at *m*/*z* 191.056 and 179.035, consistent with established MS/MS fragmentation of caffeoylquinic acids [22,23].

Coumarin derivatives exhibited neutral losses of CH_3_ and CO groups, supporting their tentative annotation in accordance with previously reported LC–MS fragmentation behavior [17].

Several flavonoid glycosides were assigned based on sequential sugar losses. A compound eluting at 19.51 min showed a precursor ion at *m*/*z* 625.1760 [M + H]^+^ and fragment ions at *m*/*z* 479.1176 and 317.0660, corresponding to losses of rhamnose (−146 Da) and glucose (−162 Da), consistent with isorhamnetin-3-rutinoside (narcissin).

Such sugar cleavage patterns and aglycone fragments are characteristic of flavonol glycosides [7,26]. Additional flavonoids were annotated based on diagnostic aglycone ions (*m*/*z* 303, 317, 287) consistent with quercetin, isorhamnetin, and kaempferol derivatives.

#### 2.1.3. Amino Acids and Derivatives (**45**–**64**)

Polar amino acids were detected mainly in positive ion mode. Tryptophan (RT 6.43 min) exhibited a precursor ion at *m*/*z* 205.0972 [M + H]^+^ with characteristic neutral losses of NH_3_ and H_2_O, consistent with reported fragmentation patterns of amino acids in LC–HRMS studies [11,16]. Other amino acids were tentatively identified based on accurate mass measurements and expected neutral losses, in line with previously described LC–HRMS metabolomics studies of plant matrices [27,28].

An Amadori-type product, N-(1-deoxy-1-fructosyl)(iso)leucine, was annotated based on accurate mass and characteristic sugar-related fragmentation, following established HRMS annotation strategies [24].

#### 2.1.4. Organic Acids (**65**–**69**)

Malic, citric, succinic, furoic, and phloretic acids were tentatively identified mainly in negative ion mode based on accurate [M − H]^−^ precursor ions and characteristic decarboxylation fragments ([M−H–CO_2_]^−^), consistent with reported LC–HRMS behavior of plant-derived organic acids [27,28]. These compounds represent common primary metabolic intermediates detected in untargeted plant metabolomics studies.

#### 2.1.5. Sulfated Compounds (**70**–**76**)

Several sulfated metabolites were detected exclusively in negative ion mode. Their tentative identification was supported by the presence of a diagnostic fragment ion at *m*/*z* 96.960, corresponding to [HSO_4_]^−^, indicative of sulfate-containing structures. Such fragmentation behavior is consistent with reported MS characteristics of sulfated flavonoids and related compounds [29,30,31]. Full structural elucidation of these metabolites would require isolation and complementary spectroscopic analysis.

#### 2.1.6. Other Compounds (**77**–**81**)

**Figure 1:**
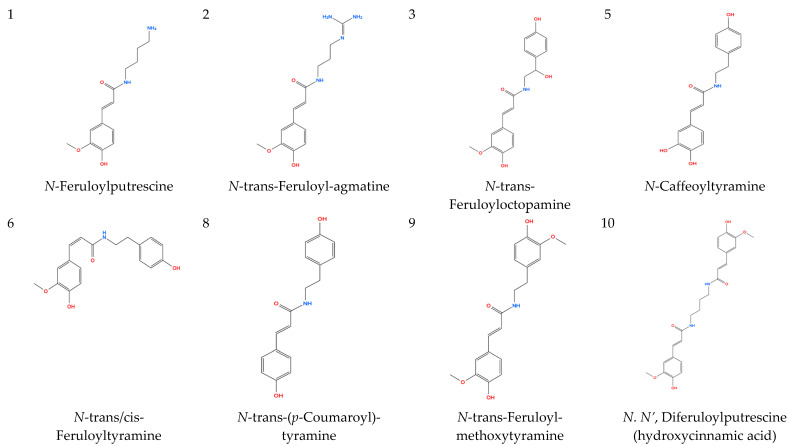

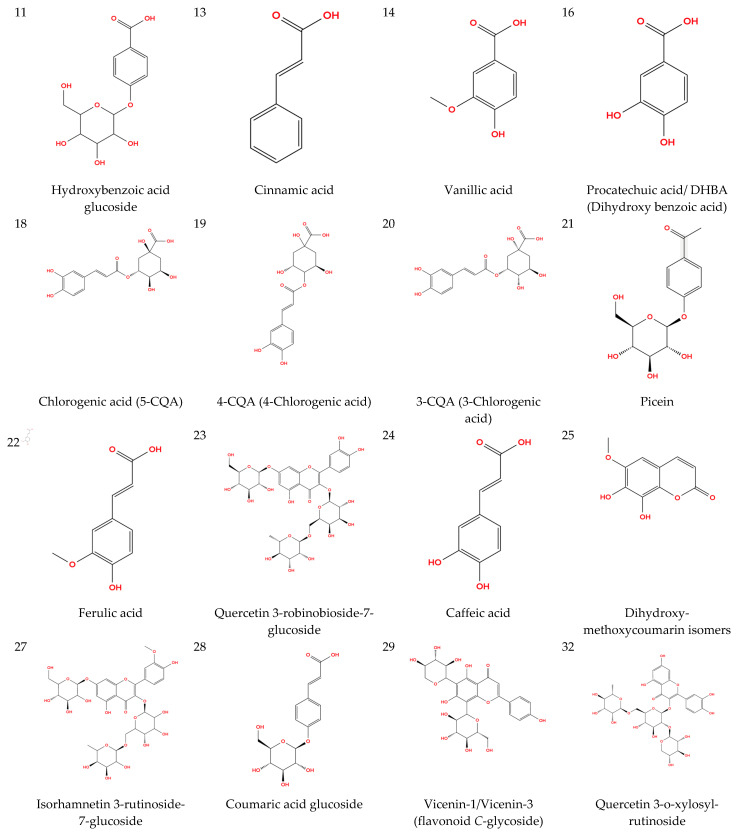

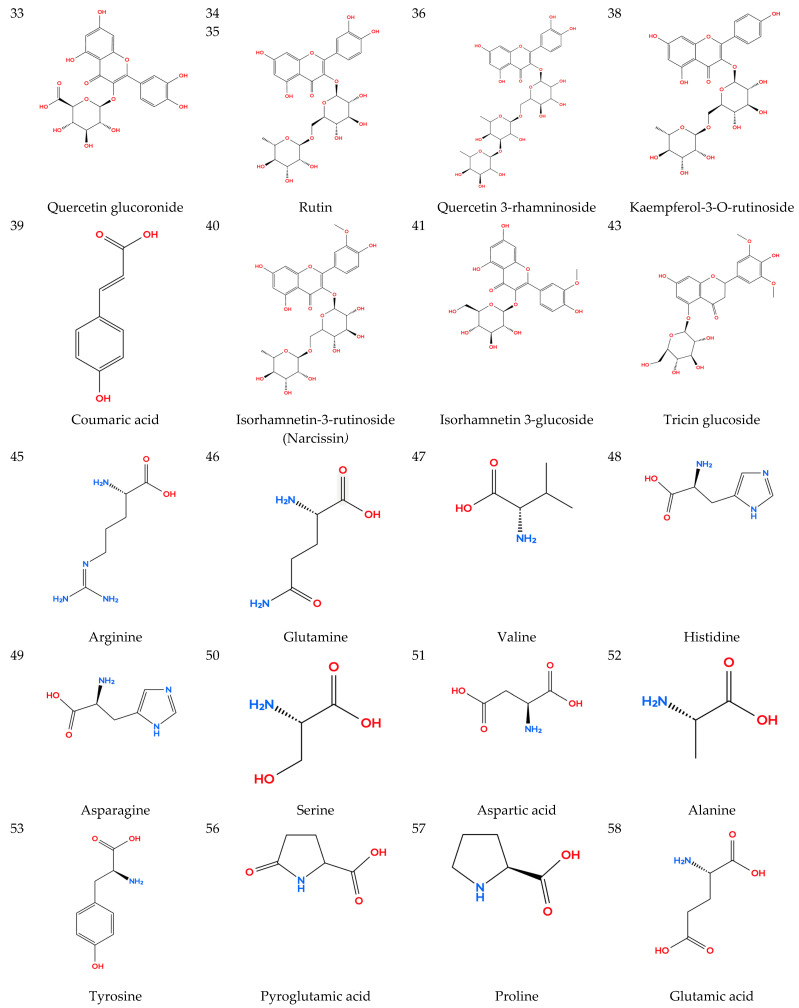

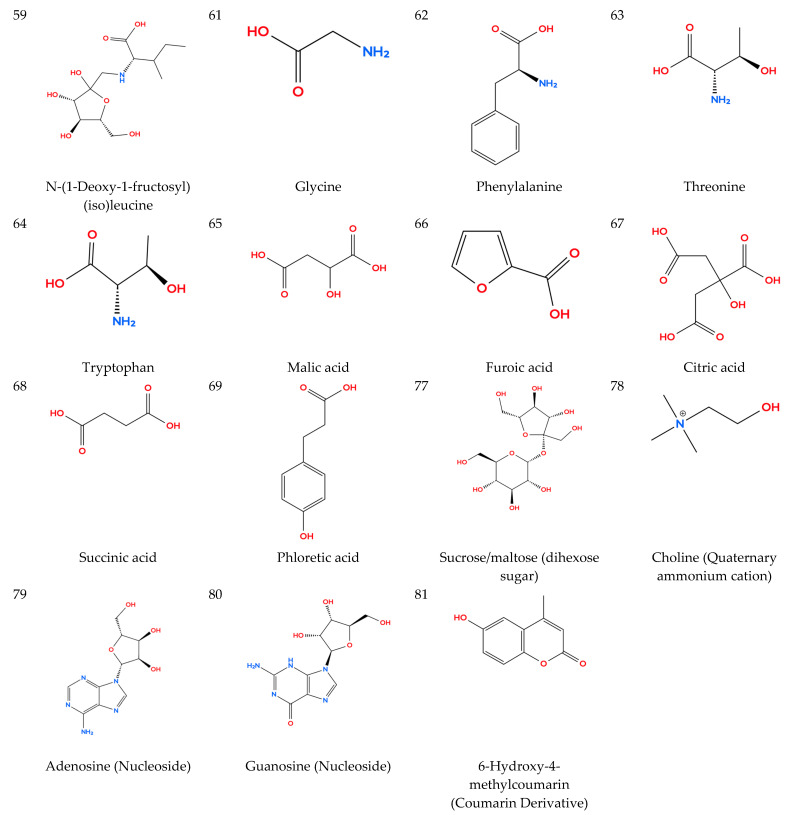
Chemical structures of selected compounds tentatively identified in the aerial parts of *Nanophyton iliense*.

Additional constituents included nucleosides, quaternary ammonium compounds, and coumarin derivatives. Adenosine and guanosine were tentatively identified based on characteristic fragment ions at *m*/*z* 136.0616 (adenine) and 152.0568 (guanine), respectively. Choline was detected as a quaternary ammonium cation at *m*/*z* 104.107. A coumarin derivative eluting at 12.30 min displayed fragmentation consistent with neutral losses of CO and CH_2_, supporting its tentative assignment. The chemical structures of the annotated compounds are presented in Figure 1.

### 2.2. Figures, Tables and Schemes

**Figure 2:**
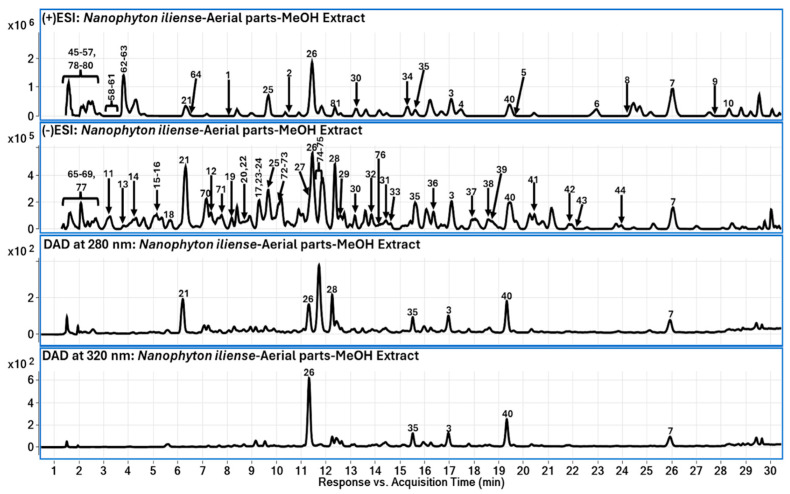
Representative LC–MS total ion chromatograms (TIC) recorded in positive and negative electrospray ionization modes and LC–DAD chromatograms at 280 nm and 320 nm for the methanolic extract of the aerial parts of *Nanophyton iliense*. Peaks are numbered according to Table 1. The 280 nm trace highlights general aromatic constituents, whereas the 320 nm profile emphasizes hydroxycinnamic acid derivatives and conjugated phenolics.

The LC–DAD and TIC-MS chromatograms shown in Figure 2 illustrate the overall metabolite distribution in the methanolic extract. The 280 nm trace highlights general aromatic constituents, whereas the 320 nm profile emphasizes hydroxycinnamic acid derivatives and conjugated phenolics. Complementary detection between positive and negative ionization modes confirms broad metabolite coverage.

### 2.3. Isolation and Structural Confirmation of Narcissin

**Figure 3:**
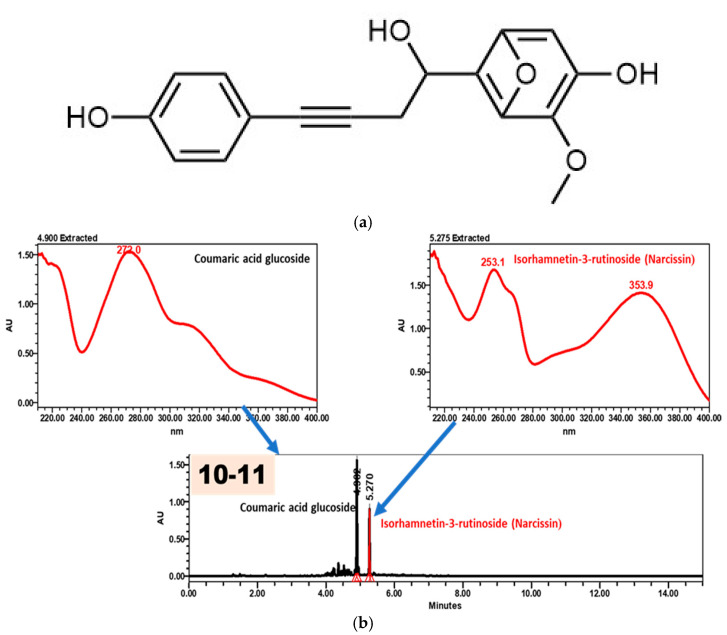
(**a**) UV–VIS spectra of coumaric acid glucoside and isorhamnetin-3-rutinoside (narcissin); (**b**) HPLC chromatogram of fraction 10–11 showing a retention time corresponding to isorhamnetin-3-rutinoside rather than coumaric acid glucoside.

In addition to LC-MS profiling, a major flavonoid was isolated from the methanolic extract. High-resolution MS data showed molecular ions at *m*/*z* 625.173 [M + H]^+^ and *m*/*z* 623.1557 [M − H]^−^, corresponding to the molecular formula C_28_H_32_O_16_. MS/MS fragmentation exhibited sequential losses of rhamnose and glucose residues, yielding an aglycone ion at *m*/*z* 317, consistent with isorhamnetin-3-rutinoside (narcissin), in agreement with previously reported flavonol glycoside fragmentation patterns [7,26]. The chromatographic behavior and UV–VIS spectra of fraction 10–11 further confirmed its identity (Figure 3).

The structure was confirmed by ^1^H and ^13^C NMR spectroscopy supported by COSY, HSQC, and HMBC correlations. Spectral data were consistent with an O-methylated flavonol glycoside structure. NMR and HR-ESI-MS spectra are provided in the Supplementary Information (Figures S1–S8).

### 2.4. Unidentified Metabolite (C_17_H_14_O_5_)

**Figure 4:**
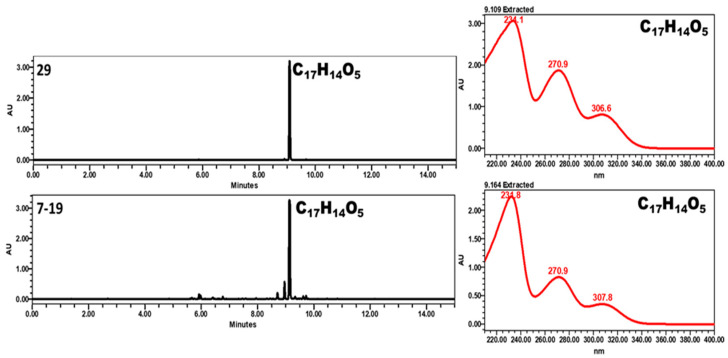
HPLC–UV chromatograms and UV spectra of fractions 29 and 7–19 showing identical retention times and UV absorption maxima, confirming the presence of the same compound (C_17_H_14_O_5_).

An additional compound with a molecular formula of C_17_H_14_O_5_ was detected in positive ion mode with molecular ions at *m*/*z* 299 [M + H]^+^ and 321 [M + Na]^+^. HPLC–UV chromatograms and UV spectra of fractions 29 and 7–19 are shown in Figure 4. The calculated double bond equivalency suggested an aromatic structure. NMR data indicated the presence of substituted aromatic rings, hydroxyl groups, and a methoxy substituent. However, despite comprehensive spectroscopic analysis, the complete structure could not be conclusively established at the current stage. Therefore, this metabolite is reported as an unidentified compound pending further structural investigation.

R = pentsubstited ring R’ = para-aryloxyphenyl.

## 3. Discussion

The comprehensive LC-DAD-QToF-MS profiling of *Nanophyton iliense* revealed a metabolite composition strongly dominated by phenylpropanoid-derived constituents, including hydroxycinnamic acid amides, flavonoids, and caffeoylquinic acid derivatives. Such chemical profiles are characteristic of stress-adapted taxa inhabiting arid and saline environments, where phenylpropanoid metabolism plays a central role in antioxidative defense and structural protection [1,2,3,4,5]. The enrichment in phenolic derivatives observed in *N. iliense* is therefore consistent with ecological expectations for halophytic shrubs exposed to high solar radiation, osmotic stress, and temperature fluctuations.

Previous phytochemical investigations within the genus *N. iliense* are limited; however, phenylpropanoid amides and phenylethanol derivatives have been reported in *Nanophyton erinaceum* [8], supporting the occurrence of this biosynthetic pathway within the genus. Hydroxycinnamic acid amides are widely distributed among flowering plants [17] and are increasingly recognized as stress-associated metabolites involved in pathogen defense, cell wall reinforcement, and oxidative stress mitigation [2,3]. Their presence in *N. iliense* suggests that phenylpropanoid–polyamine conjugation represents an important biochemical adaptation mechanism in desert Amaranthaceae species.

Comparative data from other members of the Amaranthaceae family further support this interpretation. Phenolic compounds and flavonoid glycosides have been extensively documented in Amaranthaceae taxa and are often associated with bioactive potential and ecological resilience [9,32]. Similarly, halophytic plants from arid ecosystems have been reported to accumulate diverse secondary metabolites, including phenolic acids and flavonoids, as part of their adaptive metabolic strategies [1]. The qualitative similarity between the metabolite profile of *N. iliense* and those of other stress-adapted plant species suggests conservation of phenylpropanoid-centered defense systems across environmentally challenged taxa.

The detected hydroxycinnamic acid amides in *N. iliense* align with broader reports describing the distribution of such conjugates in higher plants [25] and their induction under stress conditions [2]. These compounds are biosynthetically linked to phenylpropanoid metabolism, which integrates environmental signaling with structural and antioxidative responses [2,3]. Their relatively high representation among the annotated metabolites may indicate an adaptive metabolic shift favoring conjugated phenolics under xerophytic conditions.

Flavonoid glycosides and caffeoylquinic acid derivatives identified in this study are also widely recognized as major contributors to antioxidant capacity in plants [22,23]. Comparative LC–MS-based profiling studies of medicinal and stress-adapted plants have consistently demonstrated similar dominance of flavonol glycosides and phenolic acids [28,32,33]. Therefore, the qualitative profile of *N. iliense* does not represent an isolated chemical pattern but rather fits within the broader framework of phenolic enrichment observed in environmentally resilient species.

Importantly, this study extends existing knowledge by providing the first high-resolution metabolite annotation of *N. iliense* using LC-DAD-QToF-MS. While earlier reports on related taxa were limited to isolation of individual phenolic constituents [8], the present untargeted approach allows a more comprehensive overview of both primary and secondary metabolism. The annotation strategy followed established HRMS-based confidence principles [12,24], combined with reproducible LC–MS analytical performance and computational-assisted metabolome annotation approaches. The isolation and structural confirmation of narcissin further supports the reliability of the profiling workflow.

Although the current investigation is qualitative, the diversity of annotated phenylpropanoid derivatives suggests potential biological relevance, particularly in the context of oxidative stress mitigation and ecological adaptation. Future studies should integrate quantitative metabolomics, seasonal variation analysis, and bioactivity-guided fractionation to better understand the functional significance of the detected compounds.

Hydroxycinnamic acid amides have been widely reported in higher plants, including members of Solanaceae and Amaranthaceae families, where they are associated with stress adaptation and defense mechanisms [2,21,22,25]. Similarly, flavonoid glycosides such as quercetin, kaempferol, and isorhamnetin derivatives are commonly distributed across diverse plant taxa and often serve as chemotaxonomic markers [7,25,34]. The occurrence of chlorogenic acid isomers in *N. iliense* is consistent with their widespread distribution in stress-adapted plants and halophytic species [23,24]. Sulfated flavonoids, although less common, have been previously described in selected medicinal plants and are considered specialized secondary metabolites [29,30,31].

Overall, the phytochemical profile of *Nanophyton iliense* reflects a metabolome shaped by adaptation to arid and saline habitats, characterized by enrichment in hydroxycinnamic acid amides and flavonoid conjugates. These findings contribute to the chemotaxonomic and ecological understanding of the genus and provide a foundation for further exploration of its bioactive potential.

## 4. Materials and Methods

Acetonitrile, methanol, and formic acid are of HPLC-certified grade, and water was purified using a Milli-Q system (Millipore, Bedford, MA, USA).

### 4.1. Plant Material and Extraction

Aboveground plant material of *N. iliense* (Amaranthaceae) was collected during the flowering period in July 2025 in the Ili River region of southern Kazakhstan (44°55′ N, 79°00′ E) in the Almaty district, in the foothills of the Syugatinsky Mountains of the Northern Tien Shan, from a desert community dominated by *Ephedra* and *Nanophyton*. The identification was performed by N.Z. H, professor of the Department of Biodiversity and Bioresources, Faculty of Biology and Biotechnology. The specimen is housed under collection number 5854/28 in the herbarium collection of the Department of Biodiversity and Bioresources, Faculty of Biology and Biotechnology, Al-Farabi Kazakh National University, Almaty, Kazakhstan.

The plant material was taxonomically identified by local botanists, and voucher specimens were deposited in the herbarium of the School of Pharmacy, University of Mississippi, USA. The air-dried plant material was mechanically ground to a fine powder using a laboratory mill at room temperature. Liquid nitrogen was not used, as the material was fully air-dried prior to grinding, extracted with 80% methanol (MeOH) at a plant material-to-solvent ratio of 1:10 (*w*/*v*) under constant stirring at room temperature for 48 h. The extract was filtered and concentrated under reduced pressure using a rotary evaporator at 40 °C to yield a dark brown residue. The crude extract was stored at −20 °C until further analysis. For LC-DAD-QToF-MS analysis, a 25 mg/mL solution was prepared in HPLC-grade methanol, filtered through a 0.22 µm PTFE membrane, and placed into LC vials.

### 4.2. Sample Preparation

The 25 mg/mL extract was prepared in HPLC-grade methanol, filtered, and placed into LC vials before analysis.

### 4.3. Method

Liquid chromatography-diode array detector-quadrupole time-of-flight mass spectrometry (LC-DAD-QToF-MS).

Phytochemical profiling of the aerial parts of *N. iliense* U.P. Pratov was conducted using a liquid chromatography system coupled to a diode-array detector (DAD) and a quadrupole time-of-flight mass spectrometer (LC-DAD-QToF-MS; Model G6575A, Agilent Technologies, Santa Clara, CA, USA) equipped with an electrospray ionisation (ESI) interface operating in both positive and negative ionisation modes. Chromatographic separation was achieved on an Agilent Poroshell 120 EC-C18 column (150 mm × 2.1 mm, 2.7 µm) using an Agilent 1290 LC system maintained at 40 °C. The mobile phase consisted of water (solvent A) and acetonitrile (solvent B), both containing 0.1% formic acid, delivered at a flow rate of 0.23 mL/min. The gradient program was as follows: starting with 5% B, it ramped to 15% B over 10 min, increased to 23% B over the next 15 min, followed by a gradient to 55% B over the next 8 min, and finally to 100% B within 5 min. The injection volume was 2 µL.

Nitrogen was used as the desolvation gas at 300 °C with a flow rate of 11 L/min. Additional source parameters included a nebuliser pressure of 30 psig, sheath gas temperature of 325 °C, sheath gas flow of 11 L/min, capillary voltage of 3000 V, and fragmentor voltage of 150 V. Mass spectra were acquired over a range of *m*/*z* 50–1100. Accurate mass measurements were ensured using reference mass correction with reference ions at *m*/*z* 121.0509 (protonated purine) and 922.0098 [protonated hexakis (1H,1H,3H-tetrafluoropropoxy) phosphazine; HP-921] in positive mode, and *m*/*z* 112.9856 (deprotonated trifluoroacetic acid, TFA) and 1033.9881 (TFA-adducted HP-921) in negative mode.

Samples were analysed in all-ion MS/MS mode: experiment 1 was performed at a collision energy of 0 eV, and experiment 2 at a fixed collision energy of 40 eV. Data processing, including determination of accurate mass and molecular formulae, was performed using Mass Hunter Qualitative Analysis software (Version B.07.00).

### 4.4. Isolation of Compounds

The crude methanolic extract was subjected to repeated column chromatography on silica gel (60–120 mesh, Merck). Elution was performed using gradient systems of hexane–ethyl acetate followed by chloroform–methanol mixtures of increasing polarity. Fractions were monitored by thin-layer chromatography (TLC), combined based on similar chromatographic profiles, and concentrated under reduced pressure.

Selected fractions containing major phenolic constituents were further purified using Sephadex LH-20 column chromatography with methanol as the eluent to afford purified compounds. The major flavonoid glycoside (narcissin) was obtained after multiple chromatographic steps and subjected to NMR analysis for structural confirmation.

### 4.5. NMR Spectroscopy

Nuclear magnetic resonance (NMR) spectra were acquired on a Bruker Avance III 400 MHz spectrometer operating at 400 MHz for ^1^H and 100 MHz for ^13^C nuclei. Samples were dissolved in methanol-d_4_ and DMSO-d_6_, with chemical shifts referenced to residual solvent peaks (δ = 3.31 ppm for ^1^H and 49.0 ppm for ^13^C in MeOD; δ = 2.50 ppm for ^1^H and 39.5 ppm for ^13^C in DMSO-d_6_). Spectra were processed using TopSpin 4.1 (Bruker). Two-dimensional NMR experiments (HSQC, HMBC, COSY, DEPT) were employed to confirm connectivities and substitution patterns. The ^1^H NMR spectra provided information on proton chemical shifts, multiplicity patterns, and coupling constants, allowing identification of aromatic substitution patterns and sugar proton signals. The ^13^C NMR spectra enabled assignment of the carbon skeleton and differentiation of carbonyl, aromatic, and oxygenated carbons. COSY experiments established proton–proton coupling relationships within both aglycone and sugar moieties. HSQC spectra provided direct one-bond ^1^H–^13^C correlations for assignment of protonated carbons, while HMBC experiments revealed long-range heteronuclear correlations supporting connectivity between the flavonoid core and glycosidic residues. DEPT analysis was used to distinguish CH, CH_2_, and CH_3_ carbons and confirm substitution patterns. The NMR spectra are provided in the Supplementary Information.

### 4.6. Data Processing and Compound Identification

Tentative compound identification was based on accurate mass, retention time, and MS/MS fragmentation patterns compared with existing literature and databases (MassBank, METLIN, and GNPS). Compounds were classified into major chemical groups: hydroxycinnamic acid amides, phenolic acids, flavonoids, amino acids, organic acids, and sulfated derivatives. Annotated peaks were visualised in TIC and DAD chromatograms, and spectral assignments were confirmed through NMR correlations where available. No external reference standards were used for the majority of tentatively annotated metabolites. Therefore, compound identification was based on accurate mass measurement, isotopic pattern consistency, MS/MS fragmentation behavior, and comparison with previously published literature and publicly available spectral databases. According to widely accepted HRMS-based identification confidence levels [13], the reported annotations correspond to level 2 (putatively annotated compounds), except for narcissin, which was confirmed by isolation and NMR analysis.

## 5. Conclusions

This study provides the first comprehensive qualitative phytochemical profiling of the aerial parts of *Nanophyton iliense* using LC-DAD-QToF-MS. A total of 81 metabolites were tentatively annotated, representing diverse chemical classes, with phenolic acids, flavonoids, and hydroxycinnamic acid amides constituting the predominant groups.

The results expand the current knowledge on the secondary metabolite composition of the genus *Nanophyton* and contribute to the phytochemical characterization of desert-adapted species within Amaranthaceae.

As the present work was focused on qualitative metabolite annotation rather than quantitative determination, further studies involving targeted quantification and bioactivity evaluation are required to assess the biological relevance and potential applications of the identified compounds.

In addition, the detection of an unidentified metabolite (C_17_H_14_O_5_) suggests that *N. iliense* may contain structurally uncommon constituents warranting further structural investigation.

## Figures and Tables

**Table 1:** Tentative identification of compounds in the aerial parts of *Nanophyton iliense* using LC-QToF-MS in both positive and negative ionization modes. Compounds were tentatively identified based on accurate mass, MS/MS fragmentation (when available), and comparison with literature and databases (e.g., MassBank, METLIN, GNPS, HMDB) as well as previously reported data for related plant matrices.

#	RT (min)	Compound Name	Molecular Formula	Mass	[M + H]^+^	Fragment Ions	[M − H]^−^	Fragment Ions
Hydroxycinnamic acid amides (HCAAs)								
1	8.04	*N*-Feruloylputrescine	C_14_H_20_N_2_O_3_	264.1474	265.1548 (265.1547) *	177.0547 (ferulate-related fragment ion; [C10H9O3]^+^), 149.0598, 117.0335, 89.0385	-	-
2	10.47	N-trans-Feruloyl-agmatine	C_15_H_22_N_4_O_5_	306.1692	307.1766 (307.1765)	177.0547 (ferulate-related fragment ion; [C10H9O3]^+^), 145.0285, 117.0334, 89.0384	-	-
3	17.06	N-trans-Feruloyloctopamine	C_18_H_19_NO_5_	329.1263	330.1336 (330.1336)	177.0547 (ferulate-related fragment ion; [C10H9O3]^+^), 145.0285, 117.0336, 89.0385	328.1186 (328.1190) *	-
4	17.33	Ferulic acid derivative	C_18_H_17_NO_4_	311.1158	312.1231 (312.1230)	177.0548 (ferulate-related fragment ion; [C10H9O3]^+^), 145.0284, 117.0335, 89.0384	310.1081 (310.1085)	-
5	19.69	N-Caffeoyltyramine	C_17_H_17_NO_4_	299.1158	300.1231 (300.1230)	181.0497 [C_9_H_8_O_4_+H]^+^	298.1083 (298.1085)	-
6	22.86	N-trans/cis-Feruloyltyramine	C_18_H_19_NO_4_	313.1314	314.1388 (314.1387)	177.0548 (ferulate-related fragment ion; [C10H9O3]^+^), 145.0286 [C_10_H_8_O_3_+H-CH_4_O]^+^, 117.0335 [C_10_H_8_O_3_+H-CH_4_O-CO]^+^	312.1239 (312.1241)	-
7	26.04							
8	24.17	N-trans-(p-Coumaroyl)-tyramine	C_17_H_17_NO_3_	283.1208	284.1283 (284.1281)	165.0546 [C_9_H_8_O_3_+H]^+^	282.1134 (282.1136)	-
9	27.74	*N*-trans-Feruloyl-methoxytyramine	C_19_H_21_NO_5_	343.1420	344.1495 (344.1492)	177.0546 (ferulate-related fragment ion; [C10H9O3]^+^), 145.0284, 117.0334	342.1343 (342.1347)	-
10	28.30	N. N’, Diferuloylputrescine (hydroxycinnamic acid)	C_24_H_28_N_2_O_6_	440.1947	441.2020 (441.2020)	265.1550, 177.0548 (ferulate-related fragment ion; [C10H9O3]^+^), 145.0287, 117.0336	439.1871 (439.1875)	-
Phenolic compounds								
11	3.22	Hydroxybenzoic acid glucoside	C_13_H_16_O_8_	300.0845	-	-	299.0770 (299.0772)	137.0247 [M-H-Glc]^−^
12	7.34							
13	3.81	Cinnamic acid	C_9_H_8_O_2_	148.0524	-	-	147.0453 (147.0452)	-
14	4.26	Vanillic acid	C_8_H_8_O_4_	168.0423	-	-	167.0350 (167.0350)	-
15	5.13							
16	5.01	Procatechuic acid/DHBA (Dihydroxy benzoic acid)	C_7_H_6_O_4_	154.0266	155.0339	-	153.0195 (153.0193)	-
17	9.35							
18	5.68	Chlorogenic acid (5-CQA)	C_16_H_18_O_9_	354.0951	355.1024 (355.1024)	163.0390	353.0877 (353.0878)	191.0562
19	8.13	4-CQA (4-Chlorogenic acid)						135.0452, 173.0456, 179.035, 191.0562
20	8.77	3-CQA (3-Chlorogenic acid)						179.035, 191.0562
21	6.36	Picein	C_14_H_18_O_7_	298.1053	299.1127 (299.1125)	137.0597 [M+H-Glcpyr]^+^	343.1031 (343.1035) [M+COOH]^−^	135.0452 [M-H-Glcpyr]^−^
22	8.65	Ferulic acid	C_10_H_10_O_4_	194.0579	195.0652	-	193.0508 (193.0506)	-
23	9.23	Quercetin 3-robinobioside-7-glucoside	C_33_H_40_O_21_	772.2062	773.2137 (773.2135)	465.1027, 303.0502	771.1989	301.0347 [Quercetin aglycone-H]^−^
24	9.31	Caffeic acid	C_9_H_8_O_4_	180.0423	181.0495	-	179.0347 (179.0350)	135.0542 [M-H-CO_2_]^−^
25	9.74	Dihydroxy-methoxycoumarin isomers	C_10_H_8_O_5_	208.0372	209.0446 (209.0444)	194.0211 [M+H-CH_3_]^+^, 166.0261 [M+H-CH_3_-CO]^+^, 138.0311 [M+H-CH_3_-2CO]^+^, 120.0205 [M+H-CH_3_-2CO-H_2_O]^+^, 110.0362 [M+H-CH_3_-3CO]^+^	207.0301 (207.0299)	192.0066 [M-H-CH_3_]^−^, 164.0116 [M-H-CH_3_-CO]^−^, 108.0218 [M-H-CH_3_-CO-C_2_O_2_]^−^
26	11.48							
27	11.39	Isorhamnetin 3-rutinoside-7-glucoside	C_34_H_42_O_21_	786.2219	787.2293 (787.2291)	479.1187 [M+H-Glc-Rha]^+^, 317.0656 [M+H-2Glc-Rha]^+^	785.2143 (785.2146)	623.1613 [M+H-Glc]^−^, 315.0510 [M+H-2Glc-Rha]^−^
28	12.34	Coumaric acid glucoside	C_15_H_18_O_8_	326.1002	344.1341 (344.1340) [M+NH_4_]^+^	165.0547 [M+H-Glc]^+^, 123.0442 [M+H-Glc-C_2_H_2_O]^+^	325.0925 (325.0929)	163.0492 [M-H-Glc]^−^, 119.0503 [M-H-Glc-CO_2_]^−^
29	12.59	Vicenin-1/Vicenin-3 (flavonoid *C*-glycoside)	C_26_H_28_O_14_	564.1479	565.1551 (565.1552)	403.1049 [M+H-Glc]^+^, 271.0600 [M+H-Glc-Ara]^+^	563.1407 (563.1406)	473.1085 [M-H-90]^−^, 443.0991 [M-H-120]^−^, 269.0472 [M-H-Glc-Ara]^−^
30	13.25							
31	14.46							
32	13.80	Quercetin 3-o-xylosyl-rutinoside	C_32_H_38_O_20_	742.1946	743.2028 (743.2029)	611.1607 [M+H-Pentose], 465.1034, 303.0501 [M+H-Pentose-Rha-Glc]^+^	741.1884	300.0275 [M-H-Pentose-Rha-Glc]-
33	14.61	Quercetin glucoronide	C_21_H_18_O_13_	478.0747	479.0818 (479.0820)	303.0503 [Quercetin aglycone+H]^+^	477.0672 (477.0675)	-
34	15.19	Rutin	C_27_H_30_O_16_	610.1534	611.1609 (611.1607)	465.1048, 303.0502 [Quercetin aglycone+H]^+^	609.1459 (609.1461)	300.0274
35	15.63							
36	16.35	Quercetin 3-rhamninoside	C_33_H_40_O_20_	756.2113	757.2185 (757.2186)	303.0503	755.2043 (755.2040)	609.1423, 301.0354
37	17.94	Isorhamnetin 7-glucuronide 3-xyloside	C_27_H_28_O_17_	624.1326	625.1399 (625.1399) 647.1218 (647.1219)	493.0981 [M+H-Xyl]^+^, 317.0653 [M+H-Xyl-Glu]^+^	623.1252 (623.1254)	315.0507 [M-H-Xyl-Glu]^−^
38	18.56	Kaempferol-3-O-rutinoside	C_27_H_30_O_15_	594.1585	595.1656 (595.1657)	449.1089 [M+H-Glc]^+^ 287.0552 (aglycone)	593.1511 (593.1512)	285.0403
39	18.67	Coumaric acid	C_9_H_8_O_3_	164.0473	-	-	163.0402 (163.0401)	119.0500 [M-H-CO_2_]^−^
40	19.51	Isorhamnetin-3-rutinoside (Narcissin)	C_28_H_32_O_16_	624.1690	625.1760 (625.1763)	479.1176 [M+H-Rha]^+^, 317.0660 [M+H-Rha-Glc]^+^	623.1613 (623.1618)	315.0525 (Isorhamnetin aglycone)
41	20.42	Isorhamnetin 3-glucoside	C_22_H_20_O_12_	478.1111	479.1185 (479.1184)	317.0656 [M+H-Glc]^+^	477.1033 (477.1038)	314.0431
42	21.96	Coumarylquinic acid	C_16_H_18_O_8_	338.1002	-	-	337.0927 (337.0929)	-
43	22.08	Tricin glucoside	C_23_H_24_O_12_	492.1268	493.1340 (493.1341)	331.0812 (aglycone)	491.1191 (491.1195)	329.0671 (aglycone)
44	23.91							
Amino Acids								
45	1.45	Arginine	C_6_H_14_N_4_O_2_	174.1117	175.1191 (175.1190)	116.0705 [M+H-CH_5_N_3_ (guanidine group)]^+^, 70.0651 [M+H-CH_5_N_3_-H_2_O-CO]^+^	-	-
46	1.51	Glutamine	C_5_H_10_N_2_O_3_	146.0691	147.0765 (147.0764)	130.0497 [M+H-NH_3_]^+^, 101.0549 [M+H-H_2_O-CO]^+^	-	-
47	1.61	Valine	C_5_H_11_NO_2_	117.079	118.0861 (118.0863)	72.0808 [M+H-H_2_O-CO]^+^	-	-
48	1.75	Histidine	C_6_H_9_N_3_O_2_	155.0695	156.0769 (156.0768)	110.0711 [M+H-H_2_O-CO]^+^, 93.0446 [M+H-H_2_O-CO-NH_3_]^+^	-	-
49	1.91	Asparagine	C_4_H_8_N_2_O_3_	132.0535	133.0602 (133.0608)	87.0552 [M+H-H2O-CO]^+^, 70.0287 [M+H-H2O-CO-NH3]^+^		
50	1.99	Serine	C_3_H_7_NO_3_	105.0426	106.0497 (106.0499)	88.0393 [M+H-H_2_O]^+^	-	-
51	2.15	Aspartic acid	C_4_H_7_NO_4_	133.0375	134.0449 (134.0448)	116.0340 [M+H-H_2_O]^+^, 88.0391 [M+H-H_2_O-CO]^+^	-	-
52	2.34	Alanine	C_3_H_7_NO_2_	89.0477	90.0551 (90.0550)	-	-	-
53	2.36	Tyrosine	C_9_H_11_NO_3_	181.0739	182.0811 (182.0812)	165.0543 [M+H-NH_3_]^+^, 147.0439 [M+H-NH_3_-H_2_O]^+^, 136.0754 [M+H-H_2_O-CO]^+^, 123.0439 [M+H-NH_3_-H_2_O-CH_2_CO]^+^, 119.0491 [M+H-H_2_O-CO-NH_3_]^+^,	-	-
54	2.40	Isoleucine/leucine	C_6_H_13_NO_2_	131.0946	132.1020 (132.1019)	86.0964 [M+H-H_2_O-CO]^+^		
55	2.70							
56	2.62	Pyroglutamic acid	C_5_H_7_NO_3_	129.0426	130.0499 (130.0499)	84.0440 [M+H-CH_2_O_2_]^+^		
57	2.71	Proline	C_5_H_9_NO_2_	115.0633	116.0709 (116.0706)	70.0651 [M+H−H_2_O−CO]^+^		
58	3.04	Glutamic acid	C_5_H_9_NO_4_	147.0532	148.0603 (148.0604)	130.0497 [M+H-H_2_O]^+^, 102.0548 [M+H-H_2_O-CO]^+^, 84.0443 [M+H-2H_2_O-CO]^+^	-	-
59	3.22	N-(1-Deoxy-1-fructosyl) (iso)leucine	C_12_H_23_NO_7_	293.1475	294.1547 (294.1547)	276.1444 [M+H-H_2_O]^+^, 132.1019 [M+H-Fru]^+^, 86.0963 [M+H-Fru-CH_2_O_2_]^+^	-	-
60	3.50							
61	3.71	Glycine	C_2_H_5_NO_2_	75.032	76.0395 (76.0393)	-	-	-
62	3.80	Phenylalanine	C_9_H_11_NO_2_	165.0790	166.0863 (166.0863)	120.0807 [M+H-H_2_O-CO]^+^, 103.0540 [M+H-H_2_O-CO-NH_3_]^+^	-	-
63	3.90	Threonine	C_4_H_9_NO_3_	119.0582	120.0654 (120.0655)	102.0549 [M+H-H_2_O]^+^, 74.0600 [M+H−H_2_O−CO]^+^	-	-
64	6.43	Tryptophan	C_11_H_12_N_2_O_2_	204.0899	205.0973 (205.0972)	188.0707 [M+H-NH_3_]^+^, 170.0593 [M+H-NH_3_-H_2_O]^+^, 146.0595 [M+H-CH_2_CO]^+^	-	-
Organic acids								
65	1.68	Malic acid	C_4_H_6_O_5_	134.0215	-	-	133.0142 (133.0142)	-
66	2.03	Furoic acid	C_5_H_4_O_3_	112.0160	-	-	111.0088 (111.0088)	67.0195 [M-H-CO_2_]^−^
67	2.05	Citric acid	C_6_H_8_O_7_	192.0270	-	-	191.0199 (191.0197)	-
68	2.37	Succinic acid	C_4_H_6_O_4_	118.0266	-	-	117.0192 (117.0193)	-
69	2.68	Phloretic acid	C_9_H_10_O_3_	166.0630	-	-	165.0556 (165.0557)	-
Sulfated compounds								
70	7.07	Sulfated compound derivatives	C_13_H_18_O_9_S	350.0672	-	-	349.0593 (349.0599)	96.9599 [H_2_SO_4_-H]^−^
71	7.73		C_15_H_22_O_11_S	410.0883	-	-	409.0806 (409.0810)	377.0543 [M-H-CH_4_O]-, 96.9601 [H_2_SO_4_-H]^−^
72	10.06							
73	10.20		C_17_H_20_O_13_S	464.0625	-	-	463.0546 (463.0550)	241.0024, 96.9600 [H_2_SO_4_-H]^−^
74	11.50		C_13_H_22_O_6_S	306.1137	-	-	305.1063 (305.1064)	96.9601 [H_2_SO_4_-H]^−^
75	11.91							
76	14.26		C_12_H_22_O_9_S	342.0985	-	-	341.0907 (341.0912)	96.9600 [H_2_SO_4_-H]^−^
Others								
77	1.59	Sucrose/maltose (dihexose sugar)	C_12_H_22_O_11_	342.1162	-	-	341.1090 (341.1089)	179.0564, 161.0455, 149.0457, 89.0243, 71.0139
78	1.72	Choline (Quaternary ammonium cation)	C_5_H_14_NO^+^	104.1075	104.1071 (104.1070) * [M]^+^	86.0961 [M+H-H_2_O]^+^,	-	-
79	2.17	Adenosine (Nucleoside)	C_10_H_13_N_5_O_4_	267.0968	268.1039 (268.1040)	136.0616 (Adenine)	-	-
80	2.33	Guanosine (Nucleoside)	C_10_H_13_N_5_O_5_	283.0917	284.0990 (284.0989)	152.0568 C_5_H_5_N_5_O (Guanine)	-	-
81	12.30	6-Hydroxy-4-methylcoumarin (Coumarin Derivative)	C_10_H_8_O_3_	176.0473	177.0547 (177.0547)	163.0389 [M+H-CH_2_]^+^, 149.0596 [M+H-CO]^+^, 131.0493 [M+H-CO-H_2_O]^+^, 121.0646 [M+H-2CO]^+^	-	-

Theoretically accurate mass. Rhamnose (Rha); C_6_H_10_O_5_; Glc (hexosyl residue) = 162.0528; Xyl = 132.0423; Glu = 176.0321; C_4_H_8_O_4_ = 120.0423; C_3_H_6_O_3_ = 90.0317; CO = 27.9949; CO_2_ = 43.9898; H_2_O = 18.0106; OH = 17.0028 Da; CH_3_ = 15.0235; CH_4_ = 16.0313; NH_3_ = 17.0285. * Values in parentheses indicate theoretically calculated exact masses.

## Data Availability

The data presented in this study are available within the article and Supplementary Materials.

## Supplementary Materials

The following supporting information can be downloaded at: https://www.mdpi.com/article/10.3390/molecules31060918/s1, Figure S1: ^1^H NMR spectrum of isorhamnetin-3-rutinoside (narcissin); Figure S2: ^13^C NMR spectrum of isorhamnetin-3-rutinoside (narcissin); Figure S3: HSQC spectrum of isorhamnetin-3-rutinoside (narcissin); Figure S4: HMBC spectrum of isorhamnetin-3-rutinoside (narcissin); Figure S5: ^1^H NMR spectrum of the putative novel compound C_17_H_14_O_5_; Figure S6: ^13^C NMR spectrum of the putative novel compound C_17_H_14_O_5_; Figure S7: 2D NMR spectra (HSQC/HMBC/COSY) of the putative novel compound C_17_H_14_O_5_; Figure S8: HR-ESI-MS spectra (positive and negative modes) of the putative novel compound C_17_H_14_O_5_.

## Abbreviations

The following abbreviations are used in this manuscript:

LC-MS	Liquid Chromatography–Mass Spectrometry
LC-DAD-QToF-MS	Liquid Chromatography–Diode Array Detector–Quadrupole Time-of-Flight Mass Spectrometry
HPLC	High-Performance Liquid Chromatography
DAD	Diode Array Detector
QToF	Quadrupole Time-of-Flight
ESI	Electrospray Ionization
MS/MS	Tandem Mass Spectrometry
TIC	Total Ion Chromatogram
UV–Vis	Ultraviolet–Visible Spectroscopy
NMR	Nuclear Magnetic Resonance
HCAAs	Hydroxycinnamic Acid Amides
RT	Retention Time
*m*/*z*	Mass-to-Charge Ratio
