# Supplementary material for: Phytochemical Analysis of Plant Nanophyton iliense U.P. Pratov from Kazakhstan Using LC-MS
Source: Molecules. 2026 Mar 10;31(6):918. doi: 10.3390/molecules31060918 (PMC13029167; doi:10.3390/molecules31060918)
Supplement: Supplementary file 1 [file molecules-31-00918-s001.zip › molecules-4133965-supplementary.pdf]

Supplementary Material

Figures S1–S4.

NMR spectra (^{1}H, ^{13}C, HSQC, HMBC) of isorhamnetin-3-rutinoside (narcissin) isolated from fraction F10–11 of Nanophyton iliense.

^{1}H NMR (400 MHz, DMSO) δ 7.18 (d, *J* = 8.1 Hz, 1H), 5.91 (s, 2H), 5.23 (d, *J* = 8.0 Hz, 1H), 4.01 (q, *J* = 7.1 Hz, 0H), 3.81 (d, *J* = 3.9 Hz, 0H), 3.53 (s, 0H), 3.38 – 3.19 (m, 0H), 1.97 (s, 1H), 1.15 (t, *J* = 7.1 Hz, 1H), 0.98 (t, *J* = 6.1 Hz, 0H).

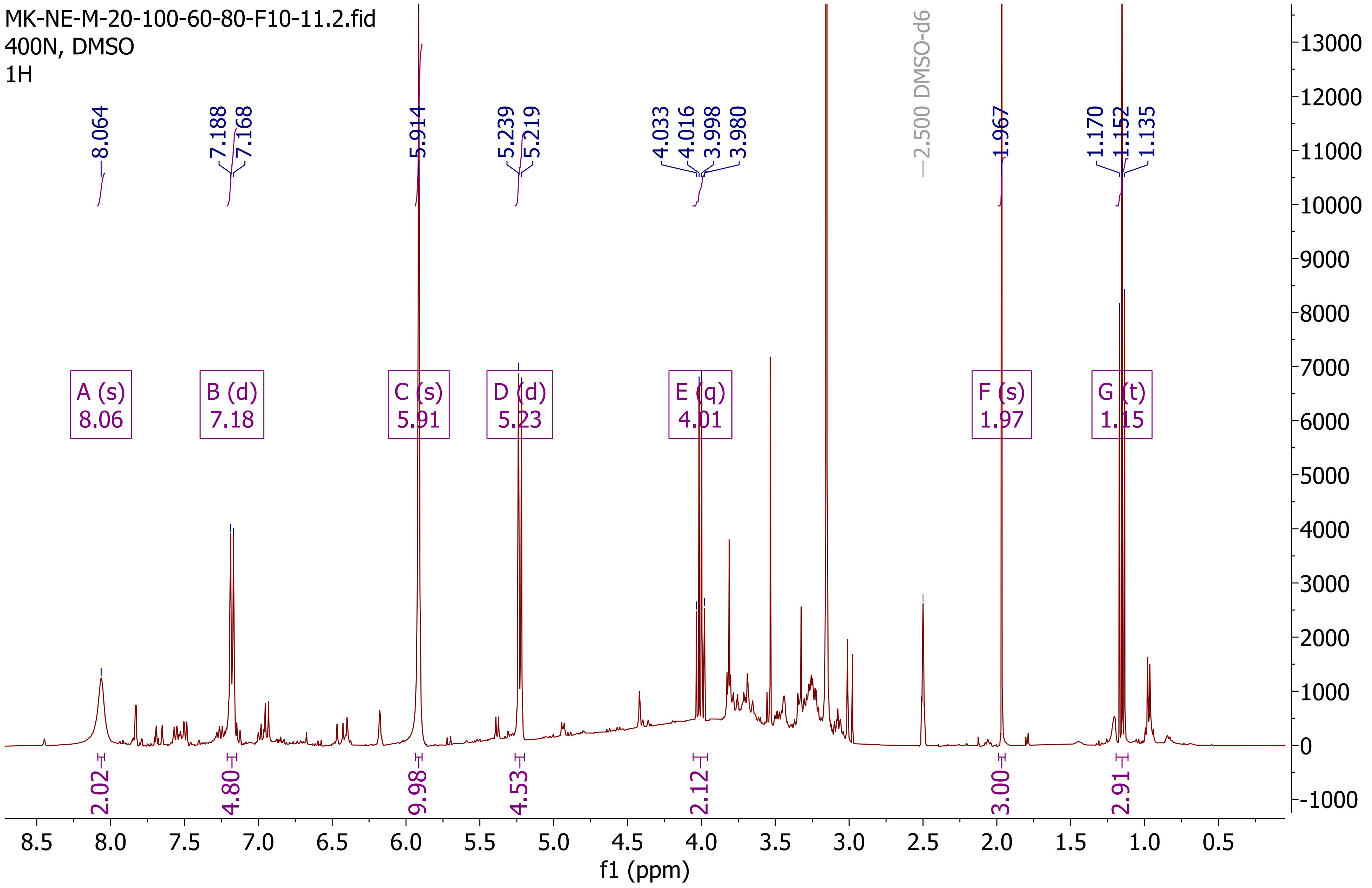

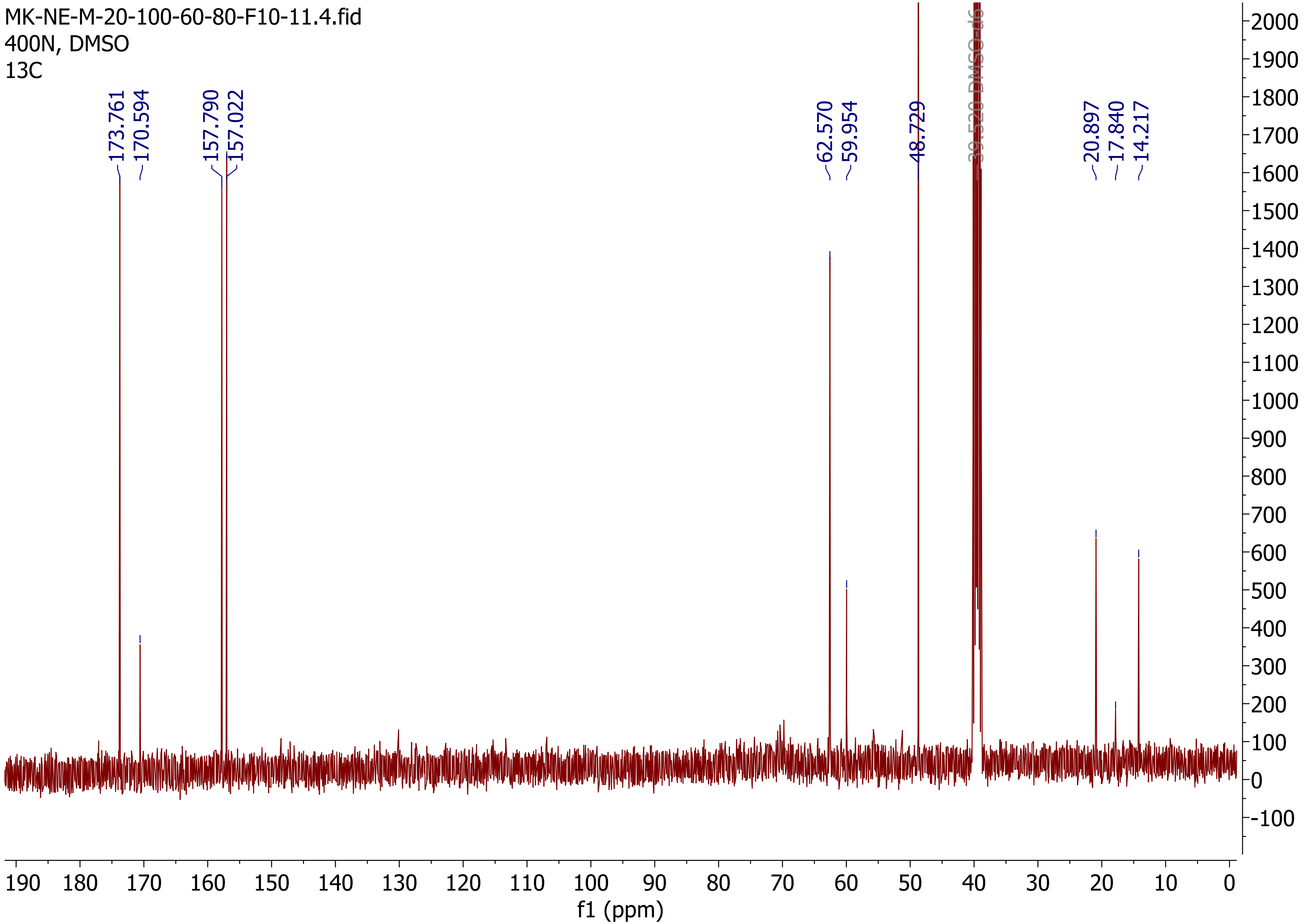
MK-NE-M-20-100-60-80-F10-11.4.fid
400N, DMSO
13C
173.761
170.594
157.790
157.022
62.570
59.954
48.729
39.520 DMSO-d6
20.897
17.840
14.217
f1 (ppm)

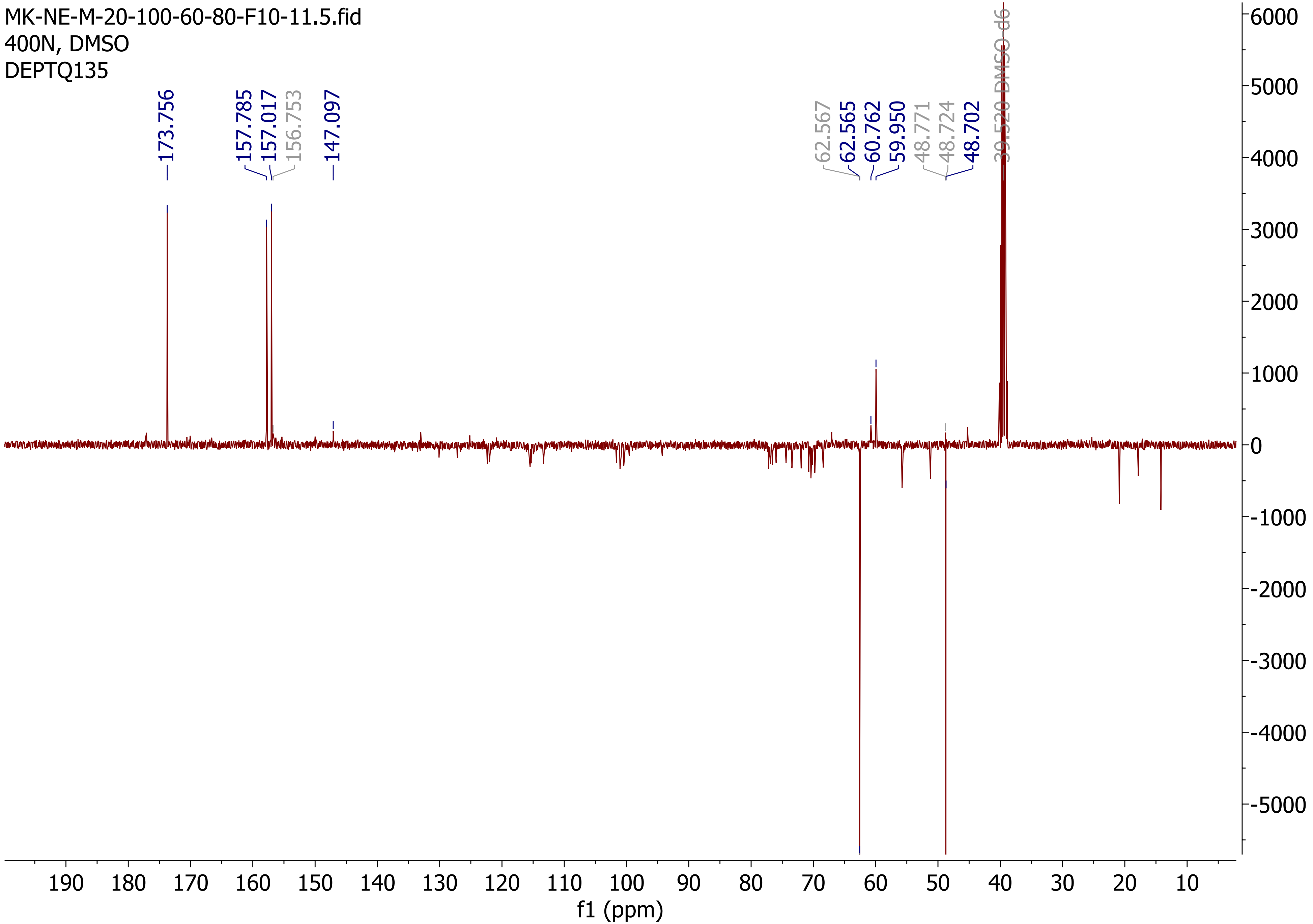
MK-NE-M-20-100-60-80-F10-11.5.fid
400N, DMSO
DEPTQ135
173.756
157.785
157.017
156.753
147.097
62.567
62.565
60.762
59.950
48.771
48.724
48.702
39.520 DMSO-d6
f1 (ppm)

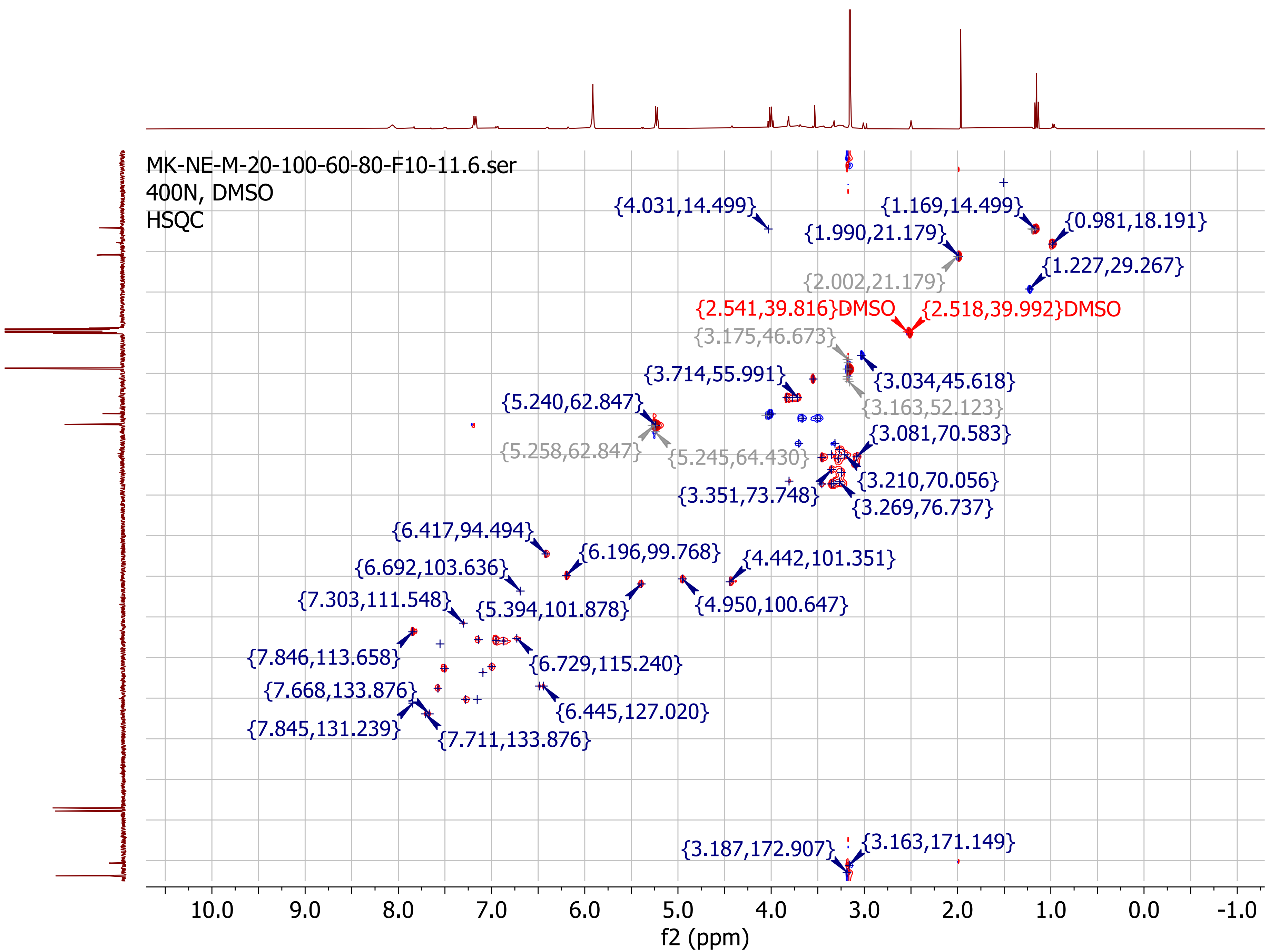
MK-NE-M-20-100-60-80-F10-11.6.ser
400N, DMSO
HSQC
{4.031,14.499}
{1.169,14.499}
{0.981,18.191}
{1.990,21.179}
{1.227,29.267}
{2.002,21.179}
{2.541,39.816}DMSO
{2.518,39.992}DMSO
{3.175,46.673}
{3.714,55.991}
{3.034,45.618}
{3.163,52.123}
{5.240,62.847}
{3.081,70.583}
{5.258,62.847}
{5.245,64.430}
{3.210,70.056}
{3.351,73.748}
{3.269,76.737}
{6.417,94.494}
{6.196,99.768}
{4.442,101.351}
{6.692,103.636}
{4.950,100.647}
{7.303,111.548}
{5.394,101.878}
{7.846,113.658}
{6.729,115.240}
{7.668,133.876}
{6.445,127.020}
{7.845,131.239}
{7.711,133.876}
{3.187,172.907}
{3.163,171.149}
f2 (ppm)
f1 (ppm)

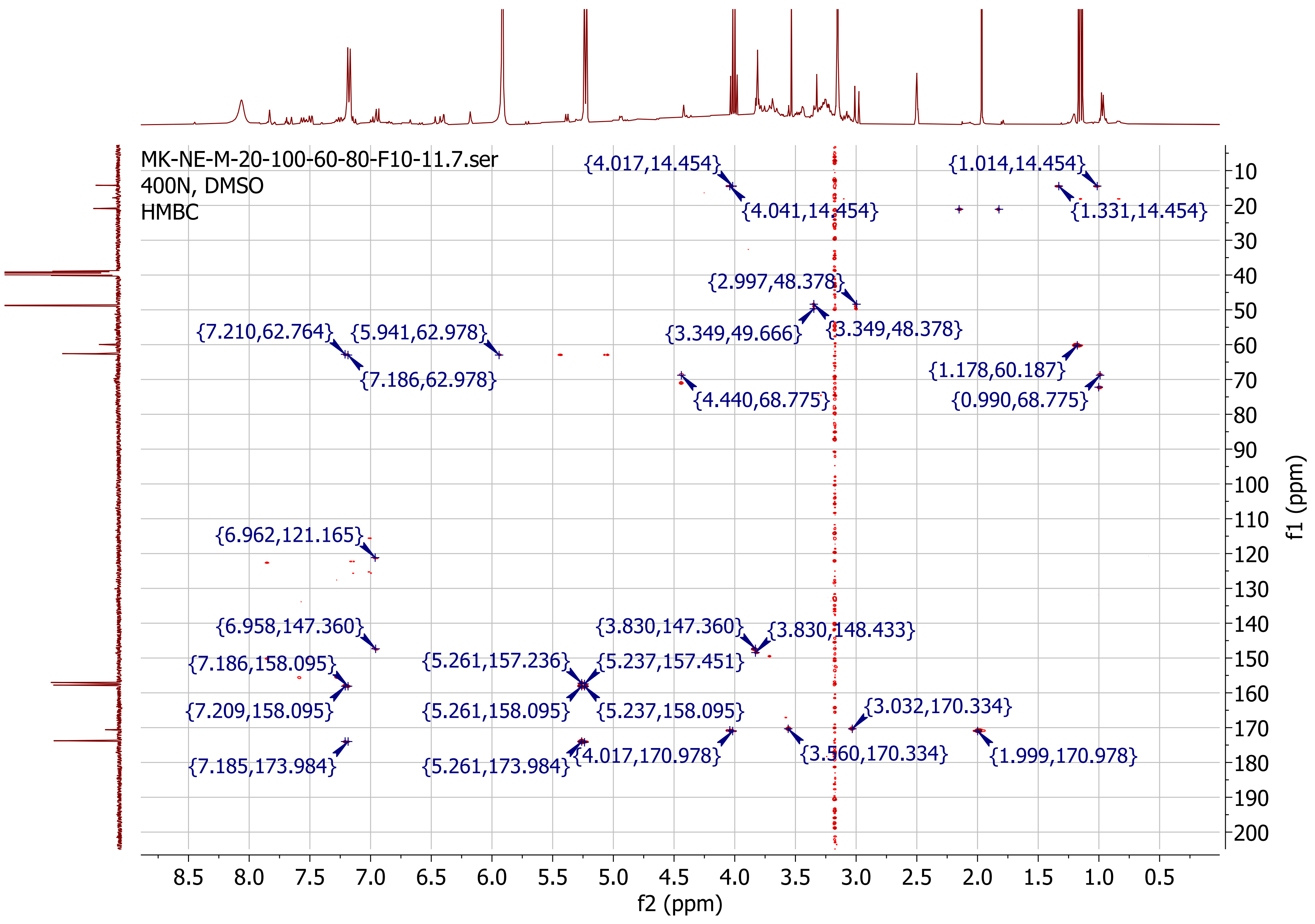

MK-NE-M-20-100-60-80-F10-11.7.ser
400N, DMSO
HMBC
{4.017,14.454}
{4.041,14.454}
{1.014,14.454}
{1.331,14.454}
{2.997,48.378}
{3.349,49.666}
{3.349,48.378}
{7.210,62.764}
{5.941,62.978}
{7.186,62.978}
{1.178,60.187}
{4.440,68.775}
{0.990,68.775}
{6.962,121.165}
{6.958,147.360}
{3.830,147.360}
{3.830,148.433}
{7.186,158.095}
{7.209,158.095}
{5.261,157.236}
{5.237,157.451}
{5.261,158.095}
{5.237,158.095}
{3.032,170.334}
{3.560,170.334}
{1.999,170.978}
{4.017,170.978}
{7.185,173.984}
{5.261,173.984}
f2 (ppm)
f1 (ppm)

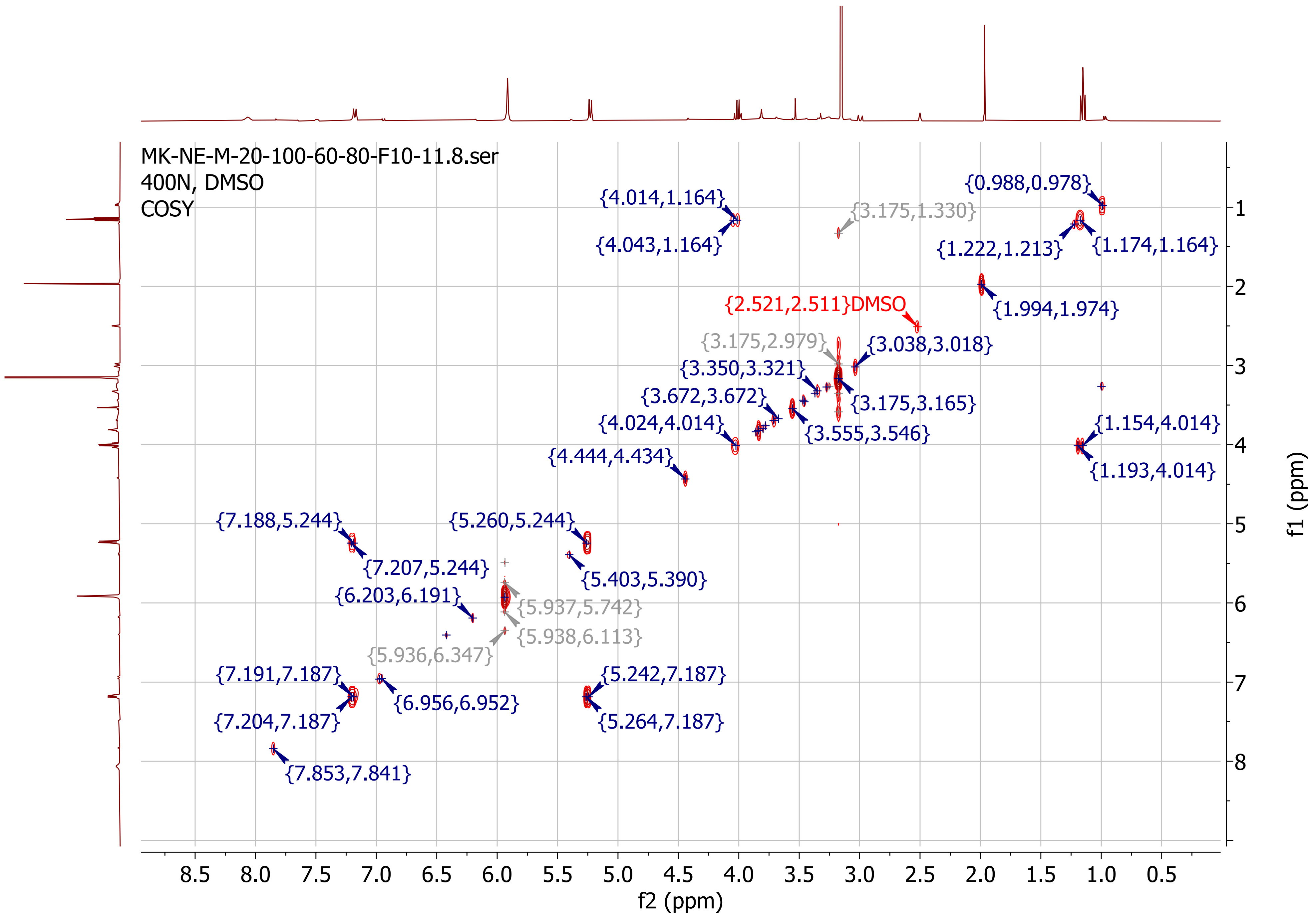
MK-NE-M-20-100-60-80-F10-11.8.ser
400N, DMSO
COSY
{4.014,1.164}
{4.043,1.164}
{3.175,1.330}
{0.988,0.978}
{1.222,1.213}
{1.174,1.164}
{1.994,1.974}
{2.521,2.511}DMSO
{3.175,2.979}
{3.038,3.018}
{3.350,3.321}
{3.672,3.672}
{3.175,3.165}
{4.024,4.014}
{3.555,3.546}
{1.154,4.014}
{4.444,4.434}
{1.193,4.014}
{7.188,5.244}
{5.260,5.244}
{7.207,5.244}
{5.403,5.390}
{6.203,6.191}
{5.937,5.742}
{5.938,6.113}
{5.936,6.347}
{7.191,7.187}
{5.242,7.187}
{6.956,6.952}
{7.204,7.187}
{5.264,7.187}
{7.853,7.841}
f2 (ppm)
f1 (ppm)

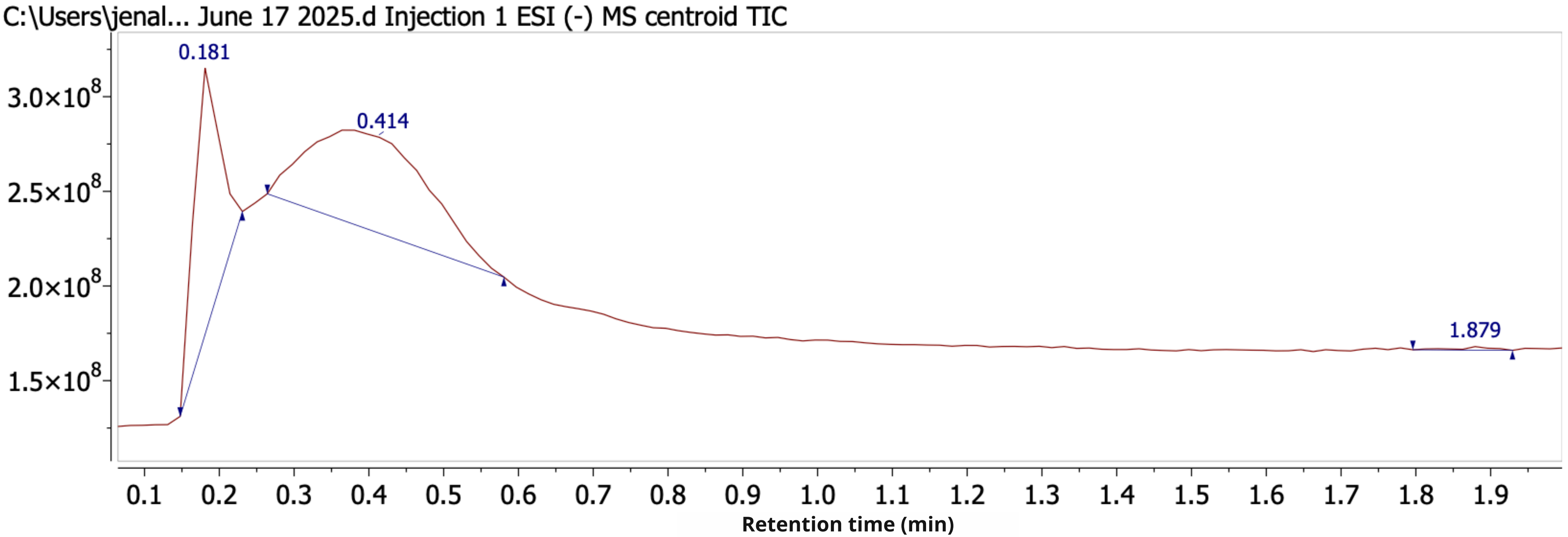
C:\Users\jenal... June 17 2025.d Injection 1 ESI (-) MS centroid TIC
0.181
0.414
1.879
3.0×10^8
2.5×10^8
2.0×10^8
1.5×10^8
0.1 0.2 0.3 0.4 0.5 0.6 0.7 0.8 0.9 1.0 1.1 1.2 1.3 1.4 1.5 1.6 1.7 1.8 1.9
Retention time (min)

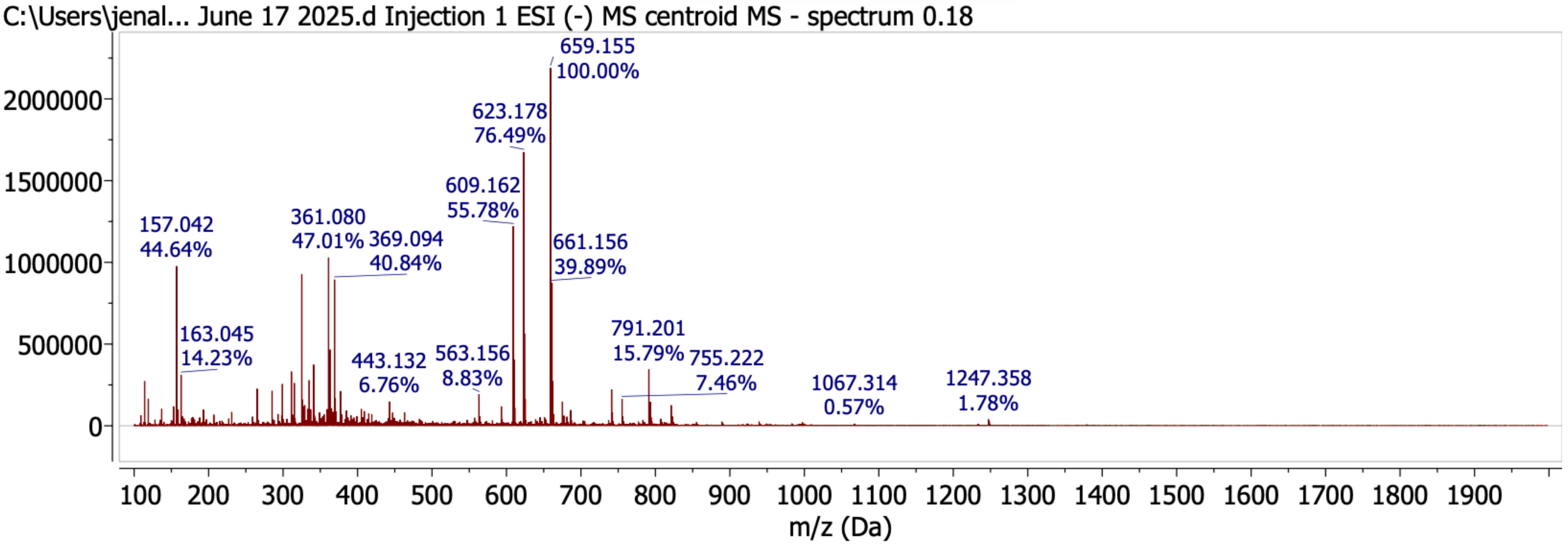
C:\Users\jenal... June 17 2025.d Injection 1 ESI (-) MS centroid MS - spectrum 0.18
659.155
100.00%
623.178
76.49%
609.162
55.78%
157.042
44.64%
361.080
47.01%
369.094
40.84%
661.156
39.89%
163.045
14.23%
443.132
6.76%
563.156
8.83%
791.201
15.79%
755.222
7.46%
1067.314
0.57%
1247.358
1.78%
2000000
1500000
1000000
500000
0
100 200 300 400 500 600 700 800 900 1000 1100 1200 1300 1400 1500 1600 1700 1800 1900
m/z (Da)

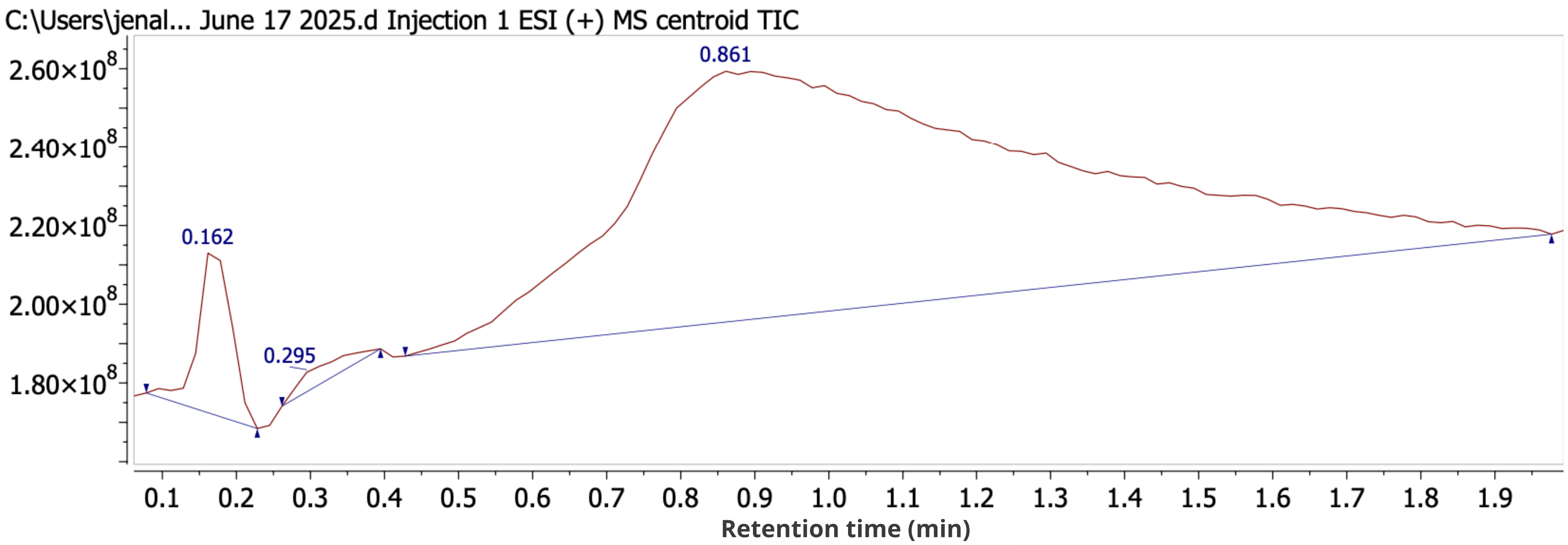

C:\Users\jenal... June 17 2025.d Injection 1 ESI (+) MS centroid TIC
0.861
0.162
0.295
Retention time (min)

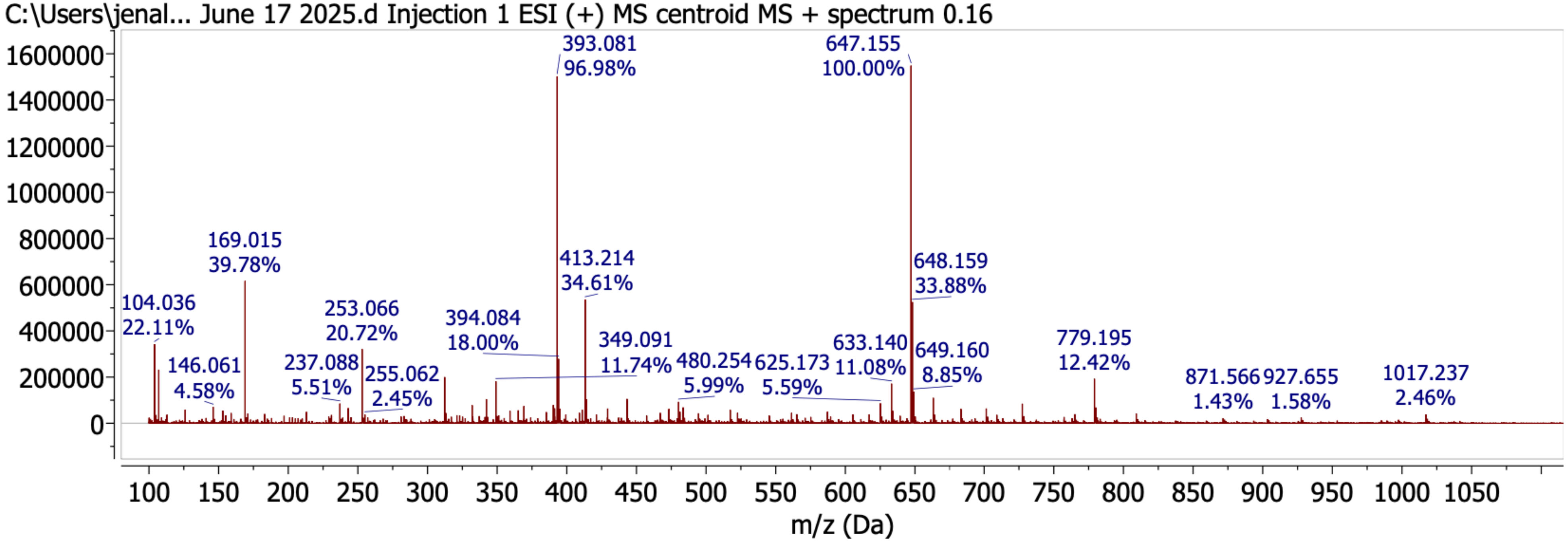

C:\Users\jenal... June 17 2025.d Injection 1 ESI (+) MS centroid MS + spectrum 0.16
393.081 96.98%
647.155 100.00%
169.015 39.78%
413.214 34.61%
648.159 33.88%
104.036 22.11%
253.066 20.72%
394.084 18.00%
349.091 11.74%
633.140 11.08%
649.160 8.85%
779.195 12.42%
146.061 4.58%
237.088 5.51%
255.062 2.45%
480.254 5.99%
625.173 5.59%
871.566 1.43%
927.655 1.58%
1017.237 2.46%
m/z (Da)

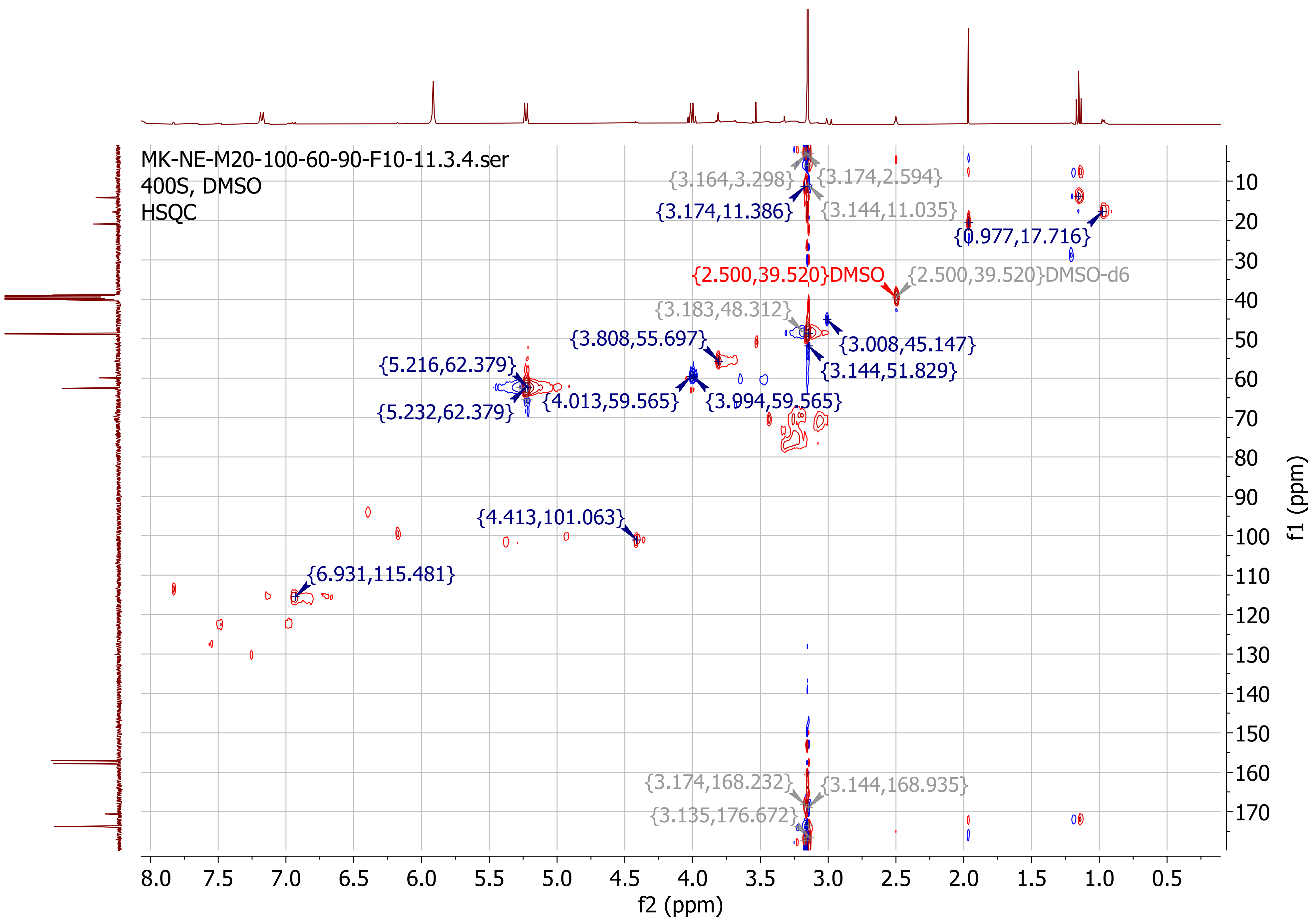
MK-NE-M20-100-60-90-F10-11.3.4.ser
400S, DMSO
HSQC
{3.164,3.298}
{3.174,2.594}
{3.174,11.386}
{3.144,11.035}
{0.977,17.716}
{2.500,39.520}DMSO
{2.500,39.520}DMSO-d6
{3.183,48.312}
{3.808,55.697}
{3.008,45.147}
{3.144,51.829}
{5.216,62.379}
{4.013,59.565}
{3.994,59.565}
{5.232,62.379}
{4.413,101.063}
{6.931,115.481}
{3.174,168.232}
{3.144,168.935}
{3.135,176.672}
f2 (ppm)
f1 (ppm)

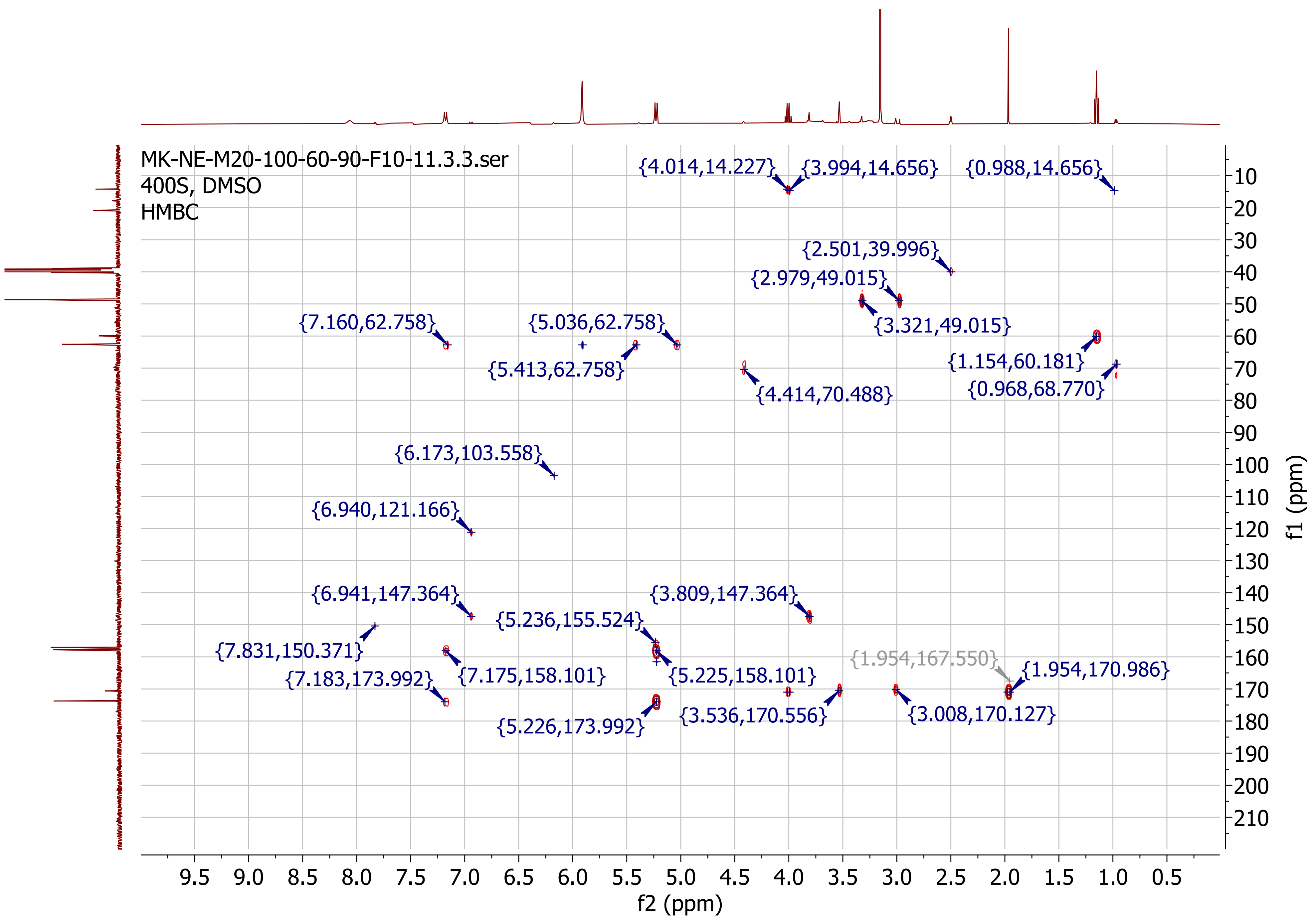
MK-NE-M20-100-60-90-F10-11.3.3.ser
400S, DMSO
HMBC
{4.014,14.227}
{3.994,14.656}
{0.988,14.656}
{2.501,39.996}
{2.979,49.015}
{3.321,49.015}
{7.160,62.758}
{5.036,62.758}
{5.413,62.758}
{1.154,60.181}
{4.414,70.488}
{0.968,68.770}
{6.173,103.558}
{6.940,121.166}
{6.941,147.364}
{3.809,147.364}
{5.236,155.524}
{7.831,150.371}
{7.175,158.101}
{5.225,158.101}
{1.954,167.550}
{1.954,170.986}
{7.183,173.992}
{5.226,173.992}
{3.536,170.556}
{3.008,170.127}
f2 (ppm)
f1 (ppm)

Figures S5–S7.

NMR spectra of the putative novel compound C17H14O5

isolated from fractions 7–19 of Nanophyton iliense.

^{1}H NMR (400 MHz, MeOD) δ 7.52 – 7.45 (m, 1H), 7.45 – 7.31 (m, 2H), 6.61 (s, 1H), 6.06 (dd, J = 15.1, 1.1 Hz, 2H), 5.47 (dd, J = 11.6, 3.2 Hz, 1H), 4.58 (s, 1H), 4.06 (s, 3H), 3.26 – 3.14 (m, 1H), 3.09 (dd, J = 16.4, 3.2 Hz, 1H), 1.29 (s, 2H).

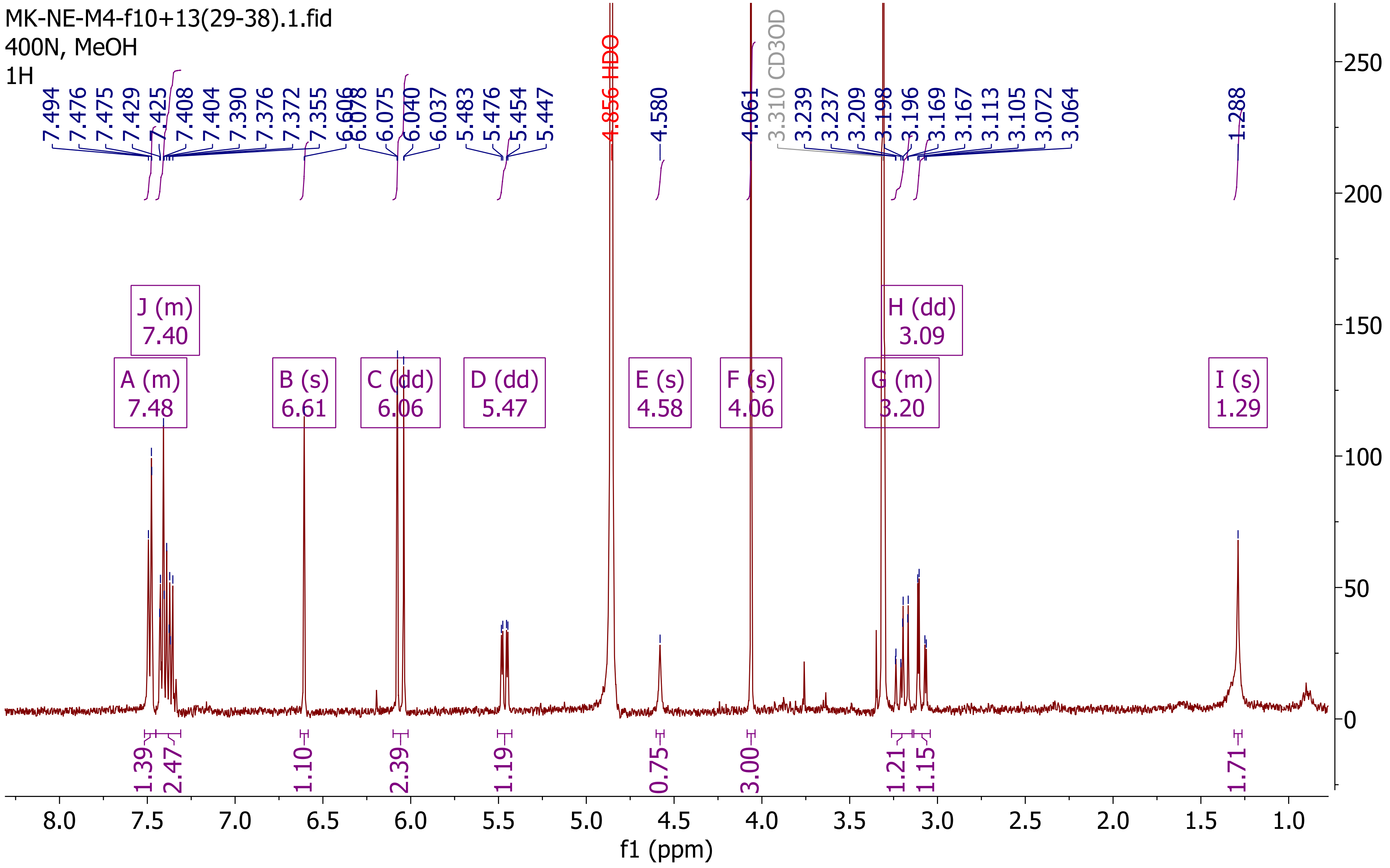

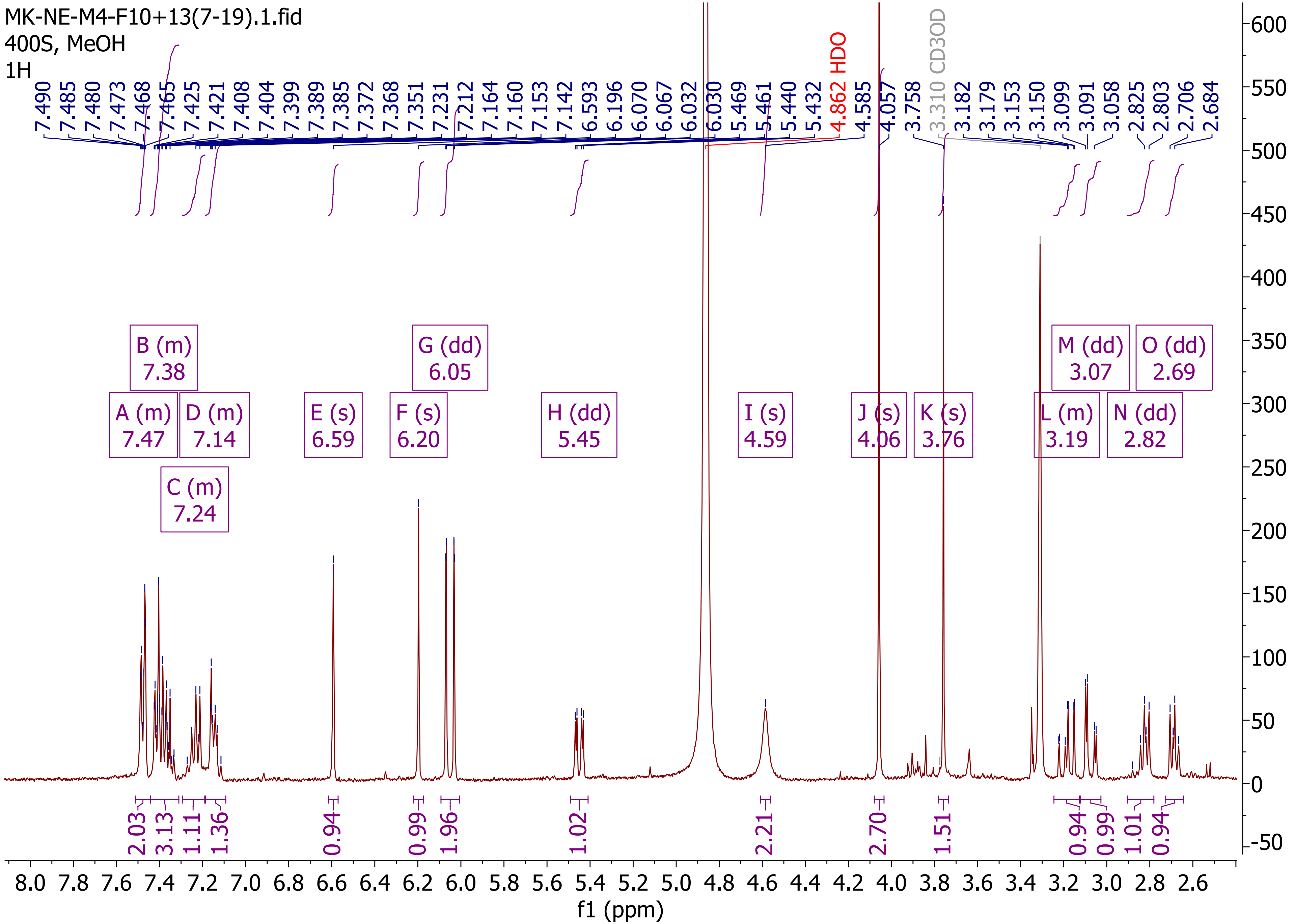

MK-NE-M4-F10+13(7-19).1.fid
400S, MeOH
1H
7.490 7.485 7.480 7.473 7.468 7.465 7.425 7.421 7.408 7.404 7.399 7.389 7.385 7.372 7.368 7.351 7.231 7.212 7.164 7.160 7.153 7.142 6.593 6.196 6.070 6.067 6.032 6.030 5.469 5.461 5.440 5.432 4.862 HDO 4.585 4.057 3.758 3.310 CD3OD 3.182 3.179 3.153 3.150 3.099 3.091 3.058 2.825 2.803 2.706 2.684
A (m) 7.47
B (m) 7.38
C (m) 7.24
D (m) 7.14
E (s) 6.59
F (s) 6.20
G (dd) 6.05
H (dd) 5.45
I (s) 4.59
J (s) 4.06
K (s) 3.76
L (m) 3.19
M (dd) 3.07
N (dd) 2.82
O (dd) 2.69
2.03 3.13 1.11 1.36 0.94 0.99 1.96 1.02 2.21 2.70 1.51 0.94 0.99 1.01 0.94
f1 (ppm)

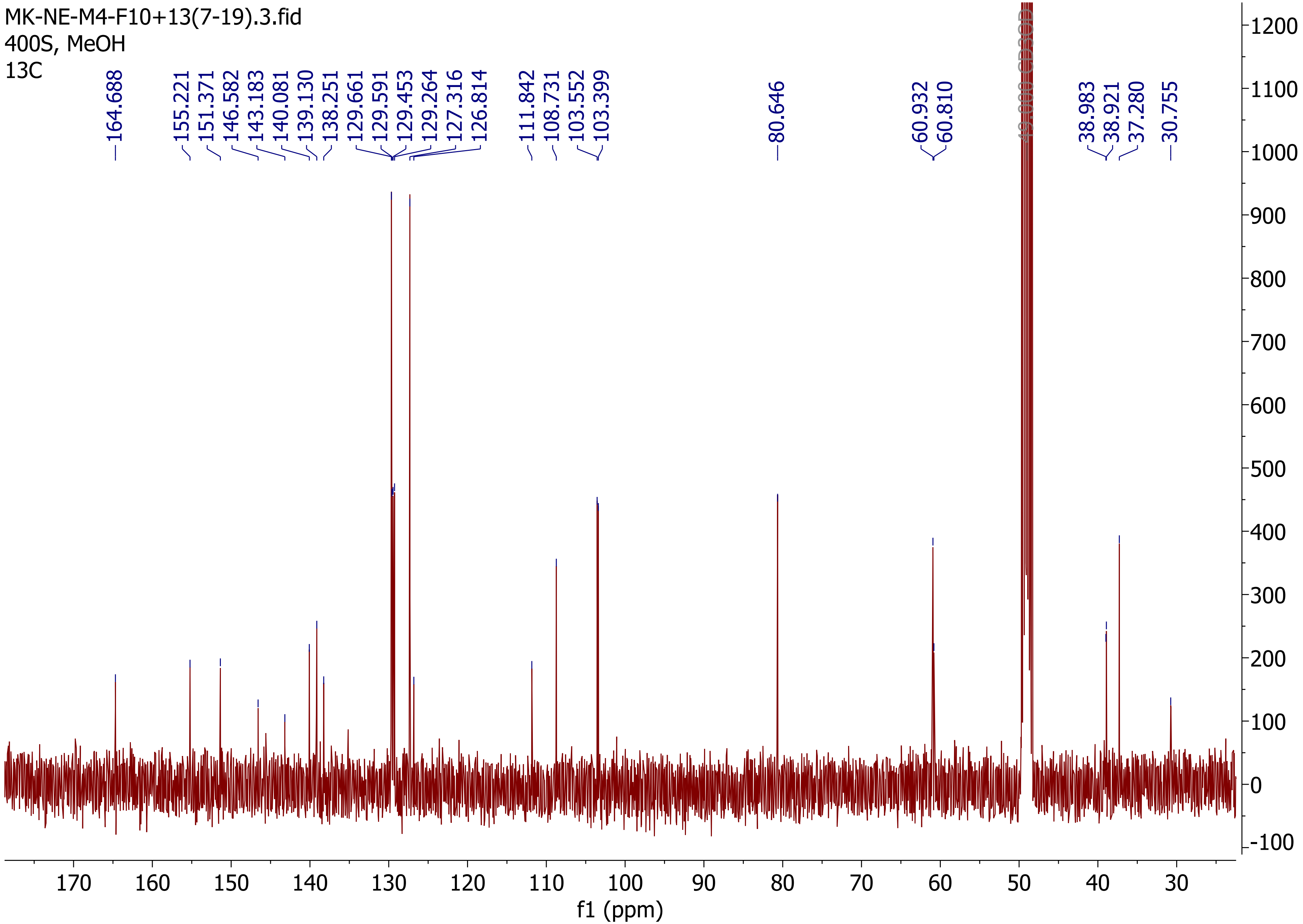
MK-NE-M4-F10+13(7-19).3.fid
400S, MeOH
13C
164.688
155.221
151.371
146.582
143.183
140.081
139.130
138.251
129.661
129.591
129.453
129.264
127.316
126.814
111.842
108.731
103.552
103.399
80.646
60.932
60.810
49.000 CD3OD
38.983
38.921
37.280
30.755
f1 (ppm)

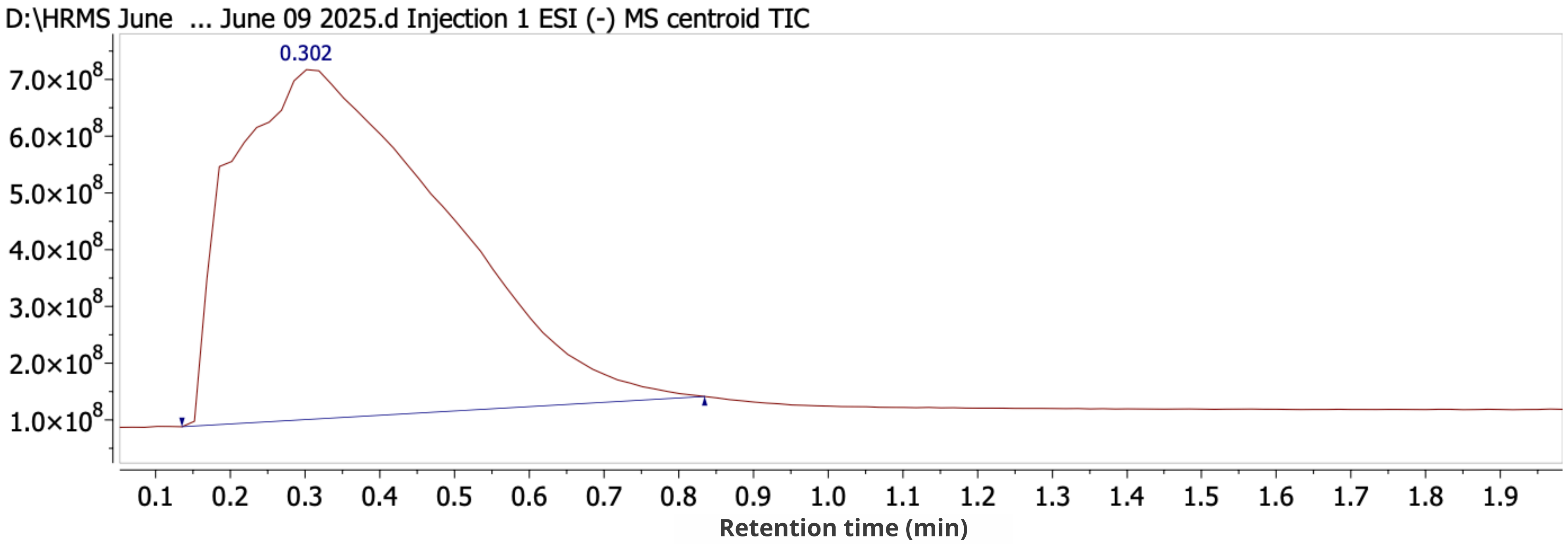
D:\HRMS June ... June 09 2025.d Injection 1 ESI (-) MS centroid TIC
0.302
7.0×10^8
6.0×10^8
5.0×10^8
4.0×10^8
3.0×10^8
2.0×10^8
1.0×10^8
0.1 0.2 0.3 0.4 0.5 0.6 0.7 0.8 0.9 1.0 1.1 1.2 1.3 1.4 1.5 1.6 1.7 1.8 1.9
Retention time (min)

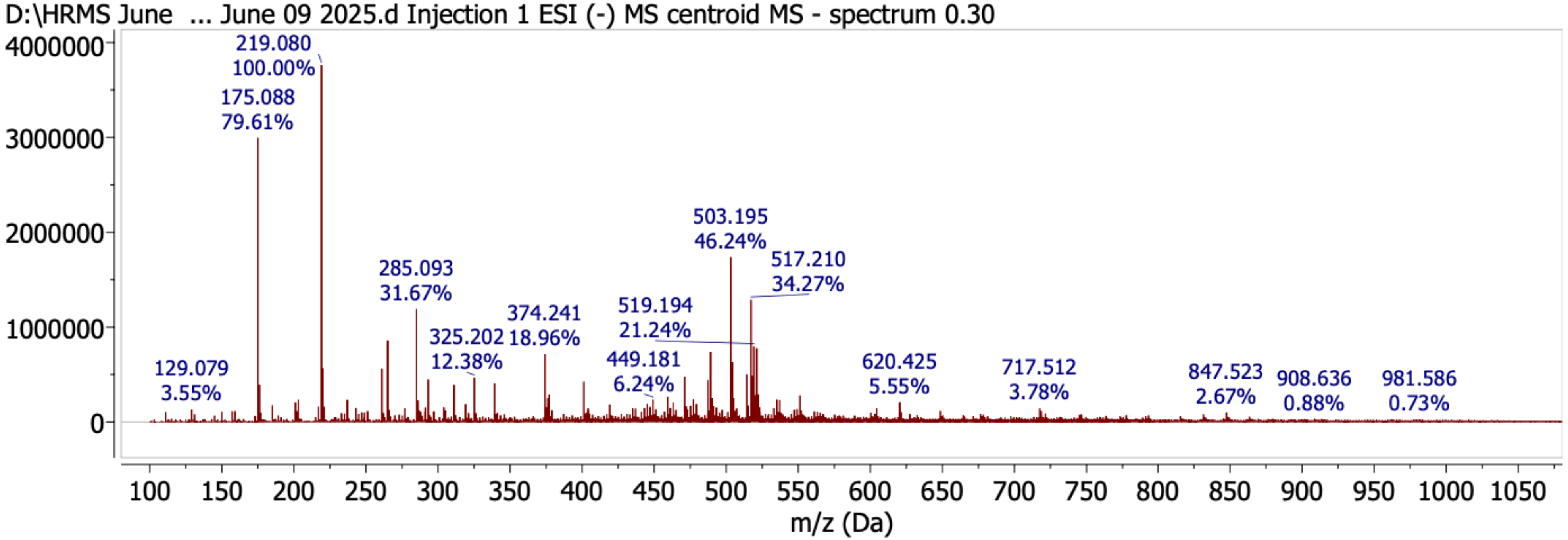
D:\HRMS June ... June 09 2025.d Injection 1 ESI (-) MS centroid MS - spectrum 0.30
4000000
3000000
2000000
1000000
0
219.080 100.00%
175.088 79.61%
285.093 31.67%
325.202 12.38%
374.241 18.96%
129.079 3.55%
449.181 6.24%
519.194 21.24%
503.195 46.24%
517.210 34.27%
620.425 5.55%
717.512 3.78%
847.523 2.67%
908.636 0.88%
981.586 0.73%
100 150 200 250 300 350 400 450 500 550 600 650 700 750 800 850 900 950 1000 1050
m/z (Da)

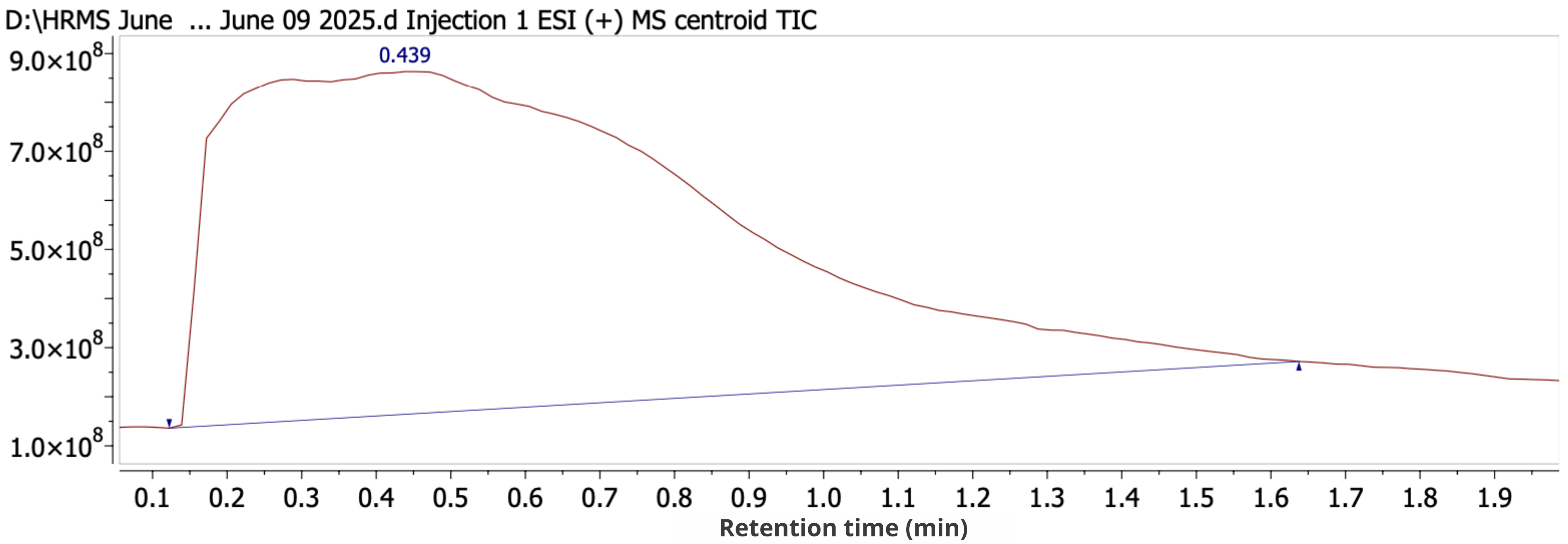

D:\HRMS June ... June 09 2025.d Injection 1 ESI (+) MS centroid TIC
0.439
Retention time (min)

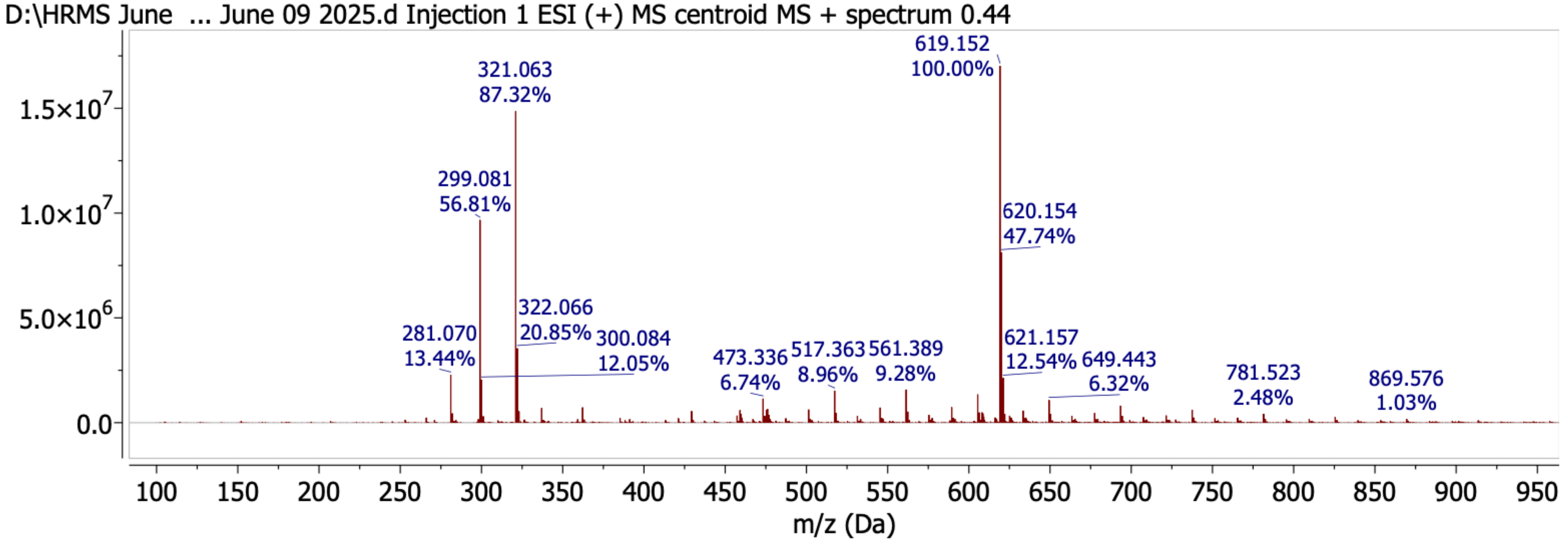

D:\HRMS June ... June 09 2025.d Injection 1 ESI (+) MS centroid MS + spectrum 0.44
619.152 100.00%
321.063 87.32%
299.081 56.81%
620.154 47.74%
322.066 20.85%
300.084 12.05%
281.070 13.44%
473.336 6.74%
517.363 8.96%
561.389 9.28%
621.157 12.54%
649.443 6.32%
781.523 2.48%
869.576 1.03%
m/z (Da)

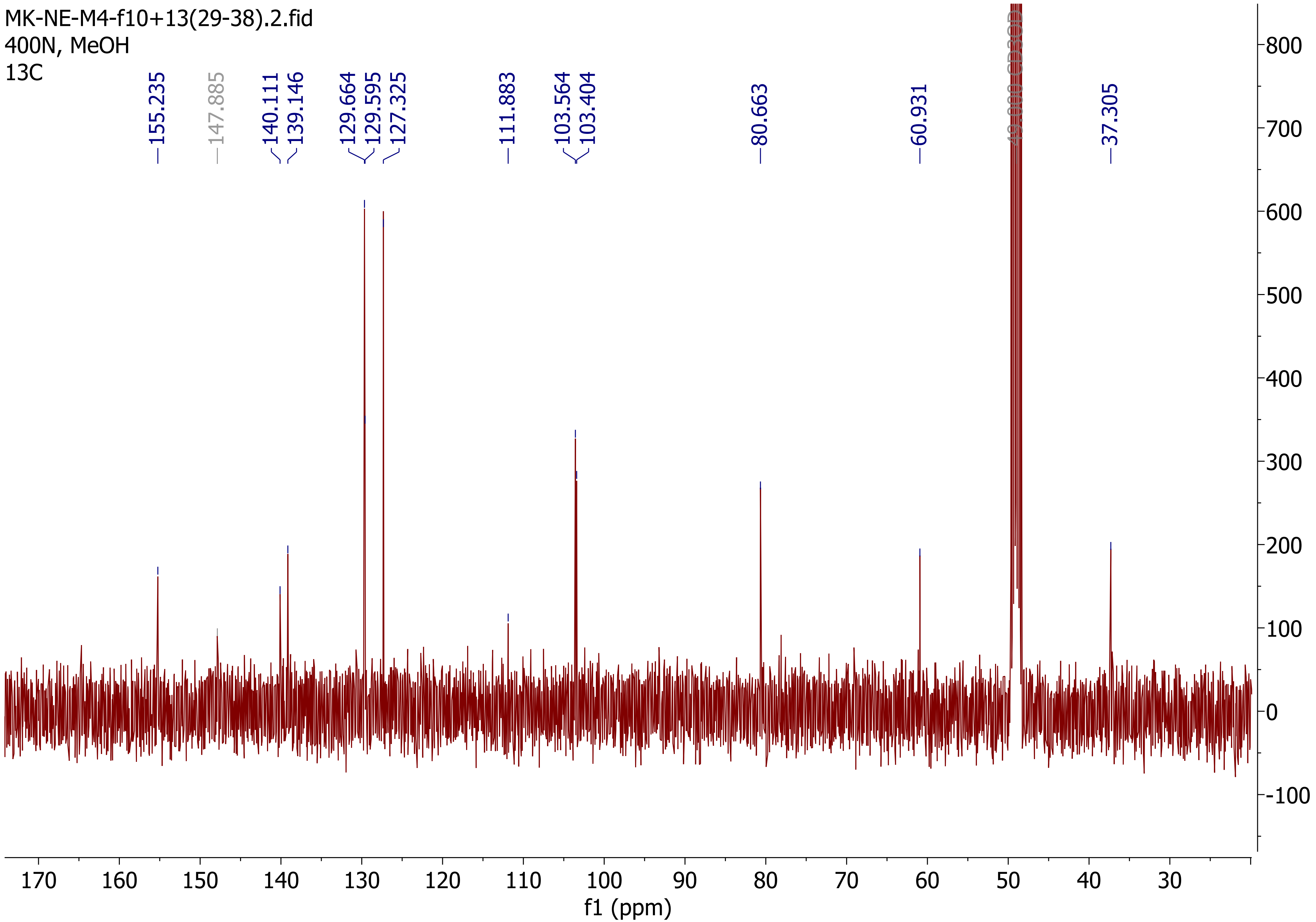
MK-NE-M4-f10+13(29-38).2.fid
400N, MeOH
13C
155.235
147.885
140.111
139.146
129.664
129.595
127.325
111.883
103.564
103.404
80.663
60.931
37.305
f1 (ppm)

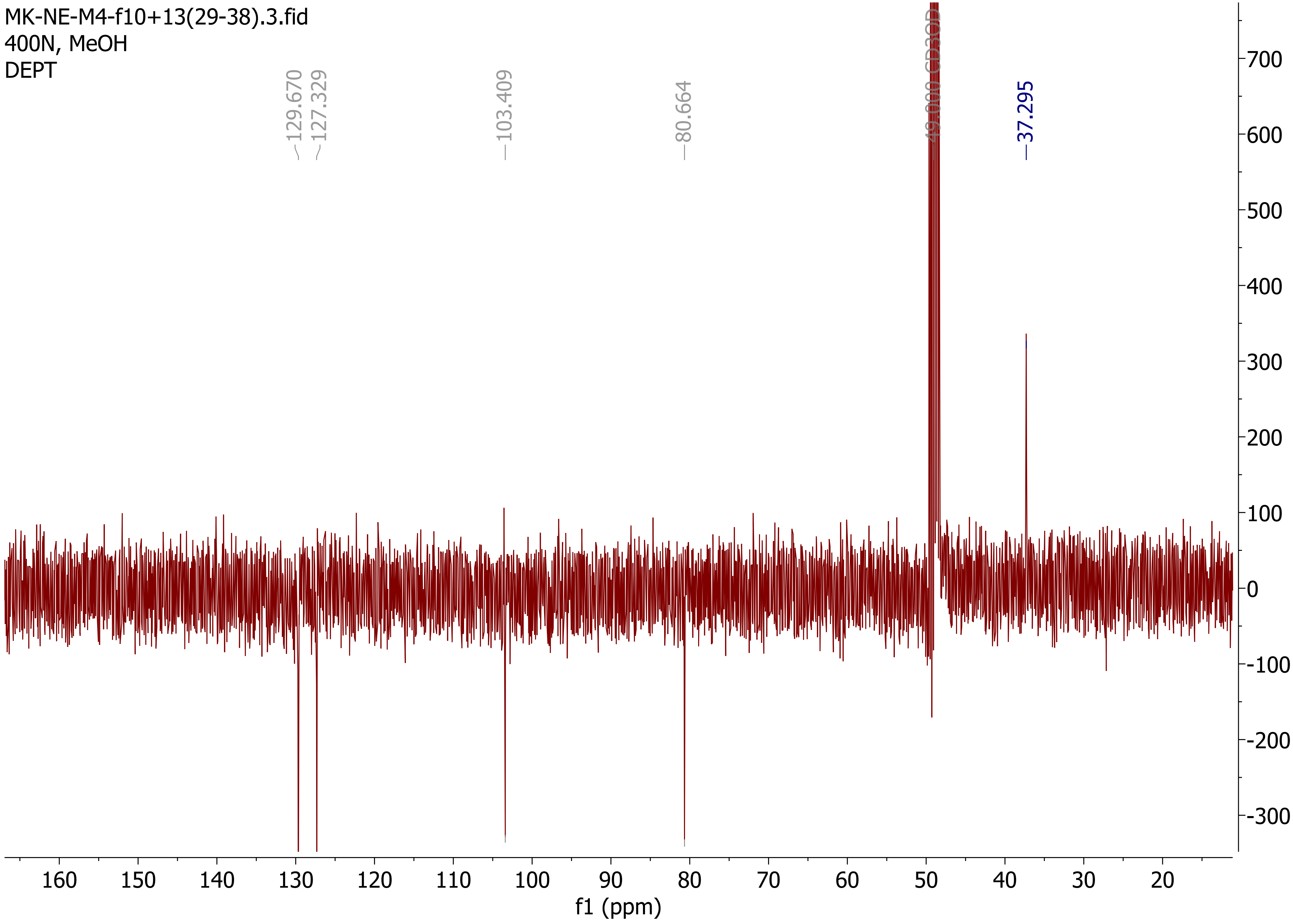

MK-NE-M4-f10+13(29-38).3.fid
400N, MeOH
DEPT
129.670
127.329
103.409
80.664
49.000 CD3OD
37.295
700
600
500
400
300
200
100
0
-100
-200
-300
160
150
140
130
120
110
100
90
80
70
60
50
40
30
20
f1 (ppm)

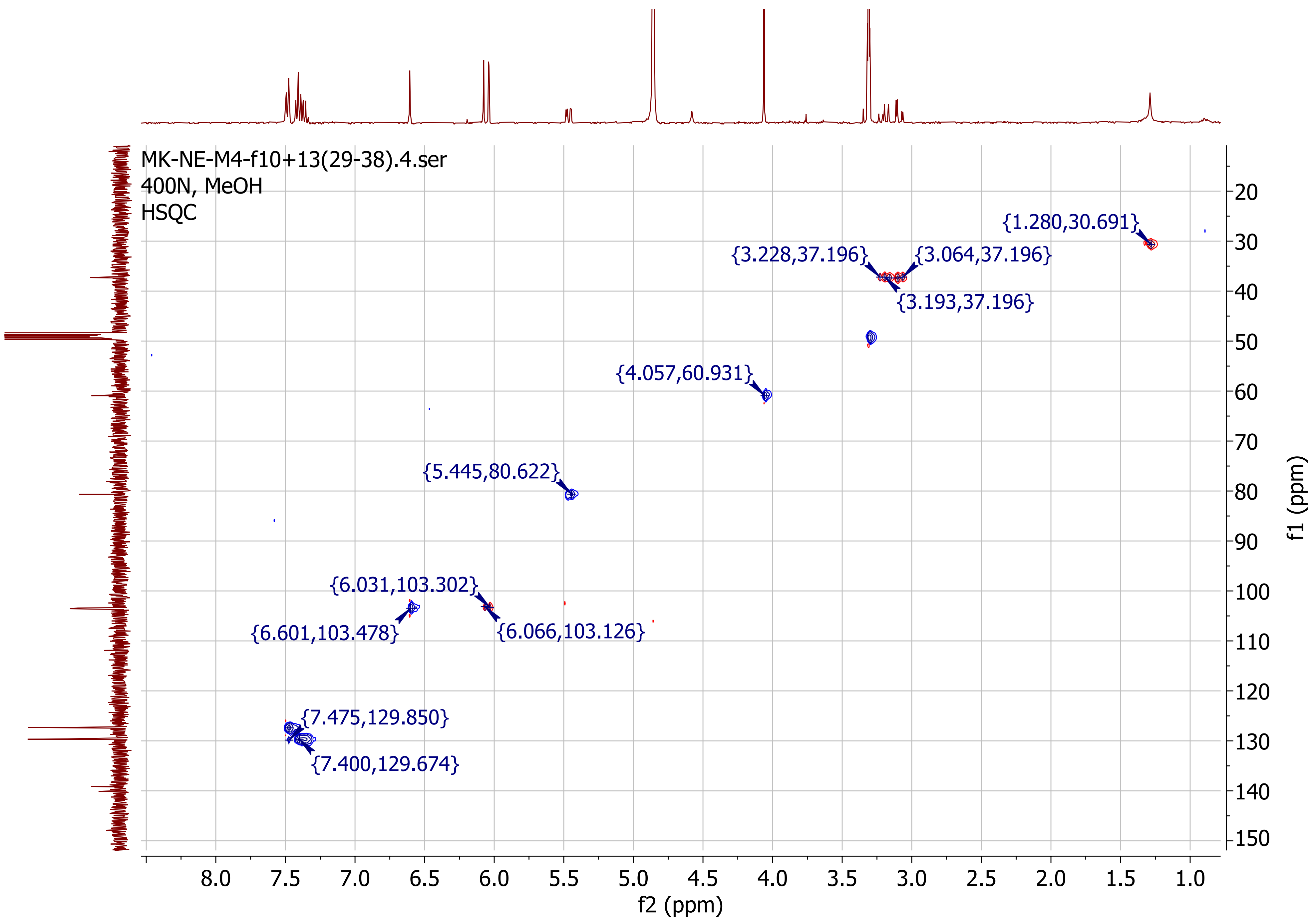
MK-NE-M4-f10+13(29-38).4.ser
400N, MeOH
HSQC
{1.280,30.691}
{3.228,37.196}
{3.064,37.196}
{3.193,37.196}
{4.057,60.931}
{5.445,80.622}
{6.031,103.302}
{6.601,103.478}
{6.066,103.126}
{7.475,129.850}
{7.400,129.674}
f2 (ppm)
f1 (ppm)

Figure S8.

High-resolution ESI-MS spectra (positive and negative modes)

of the putative novel compound $C_{17}H_{14}O_5$.

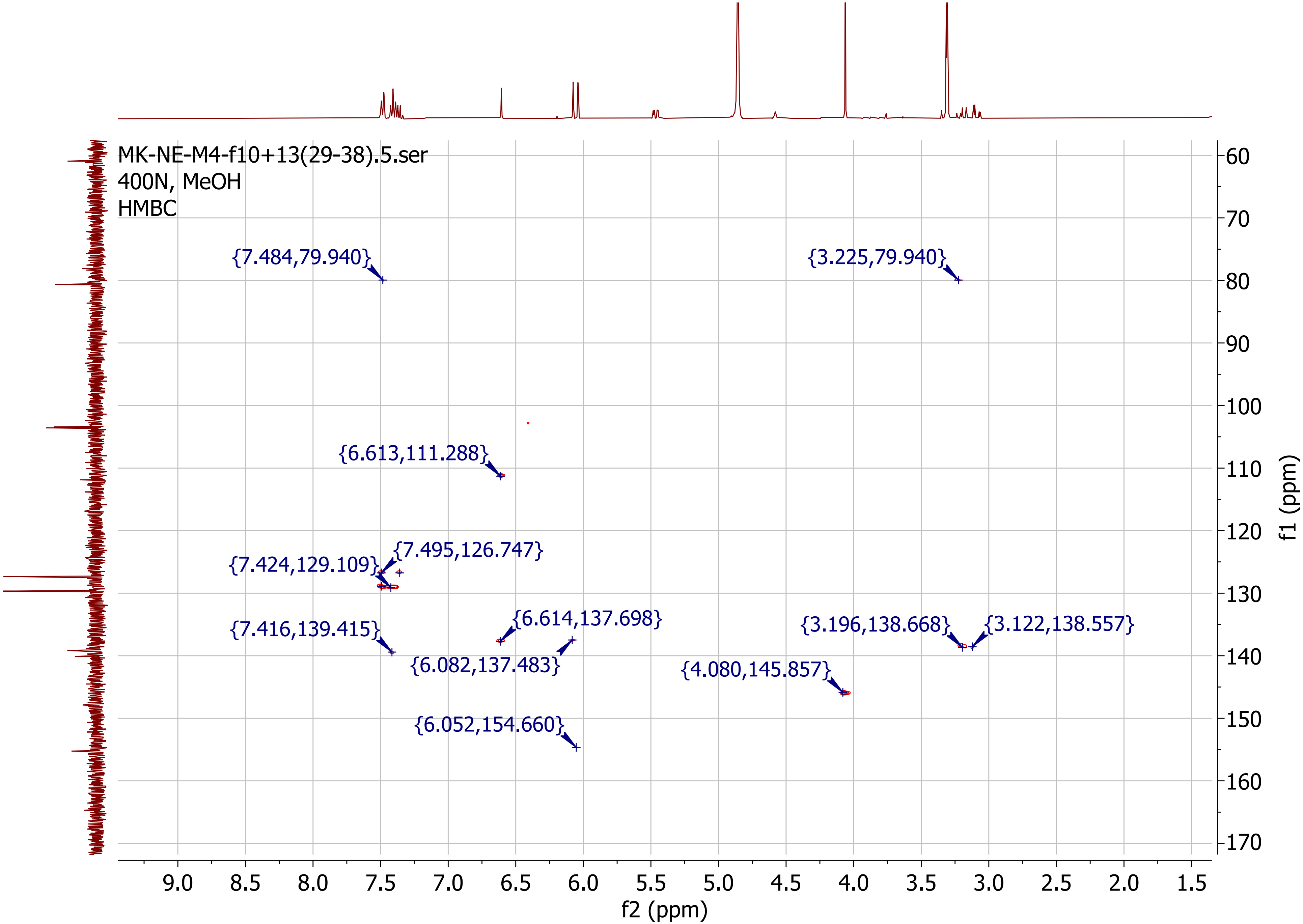

MK-NE-M4-f10+13(29-38).5.ser
400N, MeOH
HMBC
{7.484,79.940}
{3.225,79.940}
{6.613,111.288}
{7.495,126.747}
{7.424,129.109}
{6.614,137.698}
{7.416,139.415}
{6.082,137.483}
{3.196,138.668}
{3.122,138.557}
{4.080,145.857}
{6.052,154.660}
f1 (ppm)
f2 (ppm)

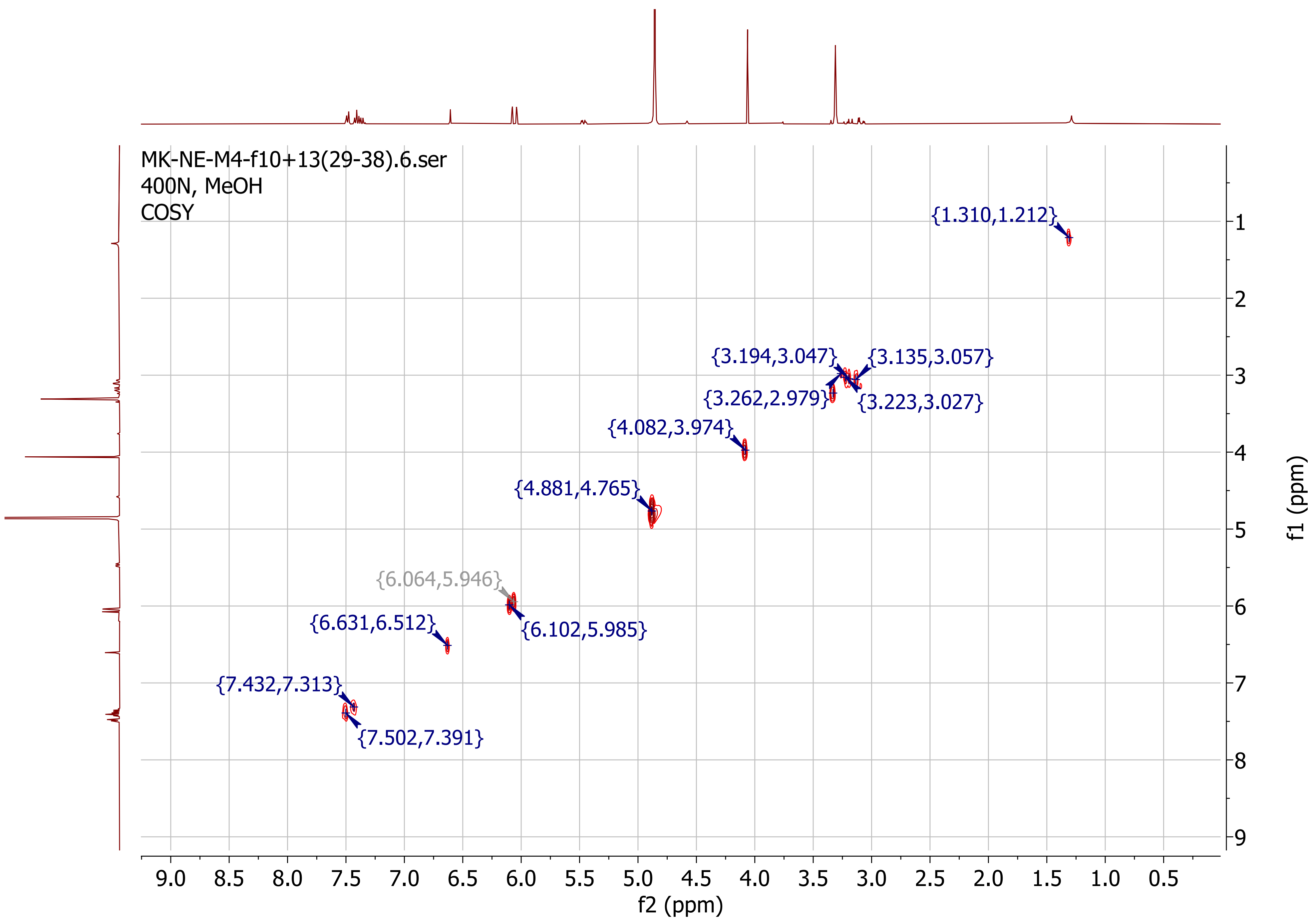
MK-NE-M4-f10+13(29-38).6.ser
400N, MeOH
COSY
{1.310,1.212}
{3.194,3.047}
{3.135,3.057}
{3.262,2.979}
{3.223,3.027}
{4.082,3.974}
{4.881,4.765}
{6.064,5.946}
{6.631,6.512}
{6.102,5.985}
{7.432,7.313}
{7.502,7.391}
f2 (ppm)
f1 (ppm)

D:\HRMS June ... June 09 2025.d Injection 1 ESI (-) MS centroid TIC

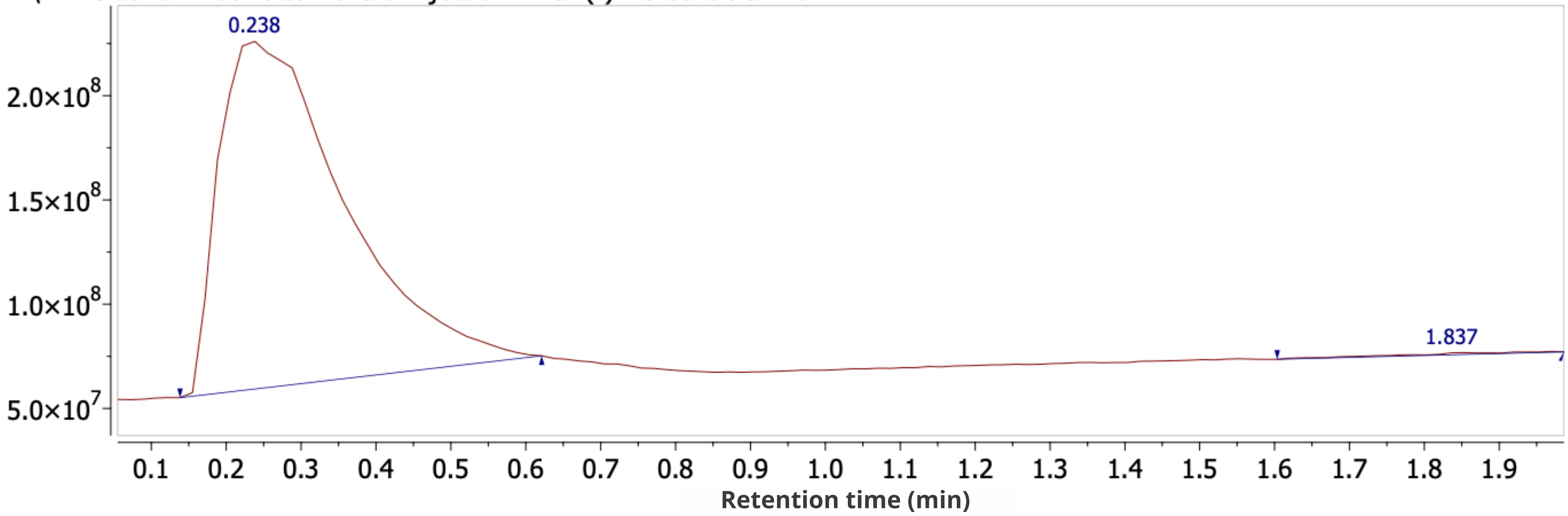

D:\HRMS June ... June 09 2025.d Injection 1 ESI (-) MS centroid MS - spectrum 0.24

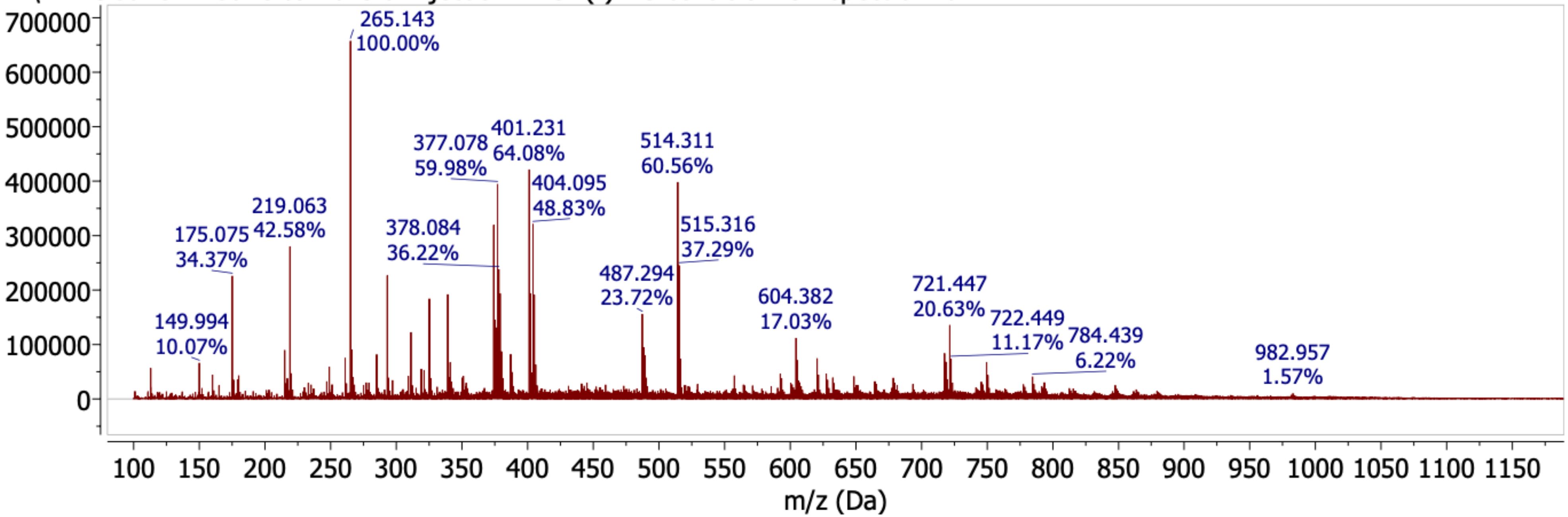

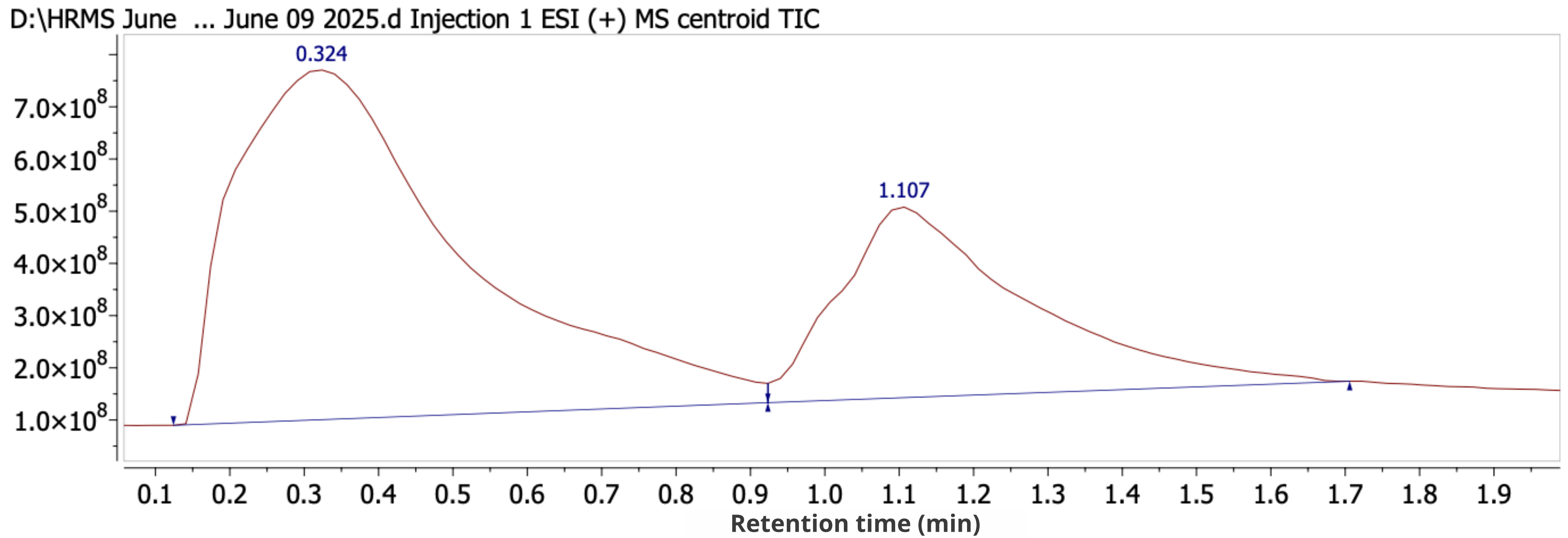
D:\HRMS June ... June 09 2025.d Injection 1 ESI (+) MS centroid TIC
0.324
1.107
7.0×10^8
6.0×10^8
5.0×10^8
4.0×10^8
3.0×10^8
2.0×10^8
1.0×10^8
0.1 0.2 0.3 0.4 0.5 0.6 0.7 0.8 0.9 1.0 1.1 1.2 1.3 1.4 1.5 1.6 1.7 1.8 1.9
Retention time (min)

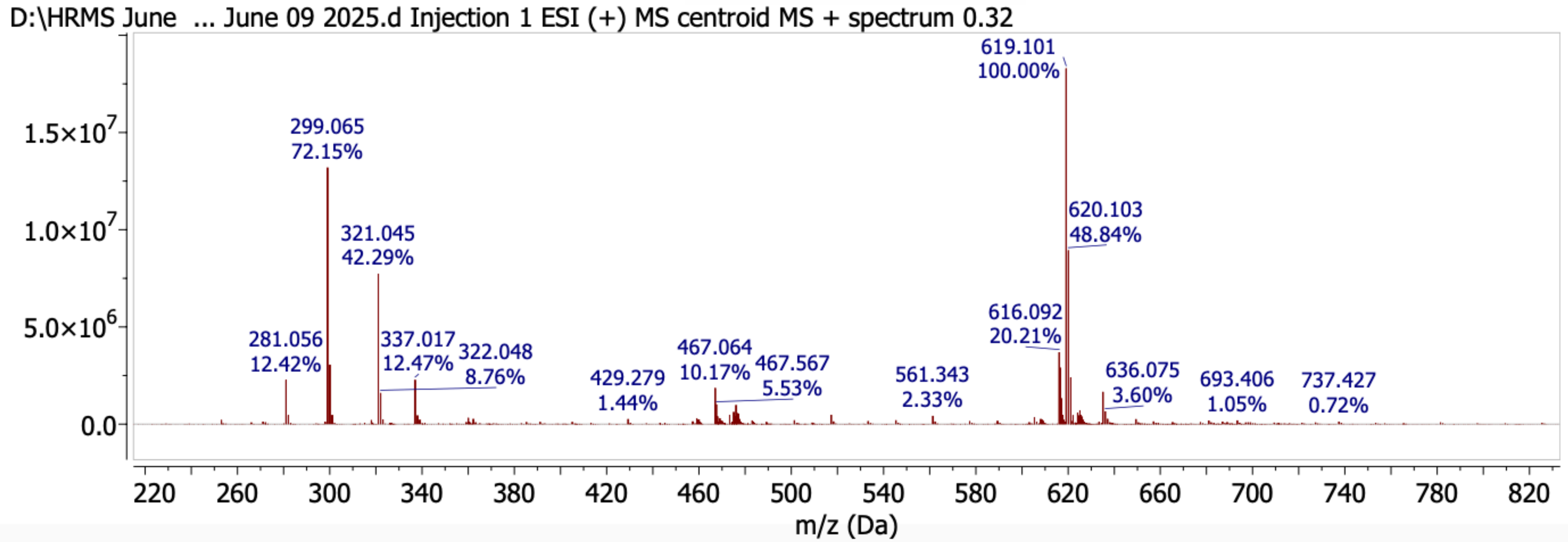
D:\HRMS June ... June 09 2025.d Injection 1 ESI (+) MS centroid MS + spectrum 0.32
619.101
100.00%
299.065
72.15%
620.103
48.84%
321.045
42.29%
616.092
20.21%
281.056
12.42%
337.017
12.47%
322.048
8.76%
429.279
1.44%
467.064
10.17%
467.567
5.53%
561.343
2.33%
636.075
3.60%
693.406
1.05%
737.427
0.72%
1.5×10^7
1.0×10^7
5.0×10^6
0.0
220 260 300 340 380 420 460 500 540 580 620 660 700 740 780 820
m/z (Da)
